# Global State Measures of the Dentate Gyrus Gene Expression System Predict Antidepressant-Sensitive Behaviors

**DOI:** 10.1371/journal.pone.0085136

**Published:** 2014-01-17

**Authors:** Benjamin A. Samuels, E. David Leonardo, Alex Dranovsky, Amanda Williams, Erik Wong, Addie May I. Nesbitt, Richard D. McCurdy, Rene Hen, Mark Alter

**Affiliations:** 1 Departments of Psychiatry and Neuroscience, Columbia University, New York, New York, United States of America; 2 AstraZeneca Pharmaceuticals, CNS Discovery, Wilmington, Delaware, United States of America; 3 Center for Neurobiology and Behavior, Department of Psychiatry, University of Pennsylvania, Philadelphia, Pennsylvania, United States of America; Karolinska Institute, Sweden

## Abstract

**Background:**

Selective serotonin reuptake inhibitors (SSRIs) such as fluoxetine are the most common form of medication treatment for major depression. However, approximately 50% of depressed patients fail to achieve an effective treatment response. Understanding how gene expression systems respond to treatments may be critical for understanding antidepressant resistance.

**Methods:**

We take a novel approach to this problem by demonstrating that the gene expression system of the dentate gyrus responds to fluoxetine (FLX), a commonly used antidepressant medication, in a stereotyped-manner involving changes in the expression levels of thousands of genes. The aggregate behavior of this large-scale systemic response was quantified with principal components analysis (PCA) yielding a single quantitative measure of the global gene expression system state.

**Results:**

Quantitative measures of system state were highly correlated with variability in levels of antidepressant-sensitive behaviors in a mouse model of depression treated with fluoxetine. Analysis of dorsal and ventral dentate samples in the same mice indicated that system state co-varied across these regions despite their reported functional differences. Aggregate measures of gene expression system state were very robust and remained unchanged when different microarray data processing algorithms were used and even when completely different sets of gene expression levels were used for their calculation.

**Conclusions:**

System state measures provide a robust method to quantify and relate global gene expression system state variability to behavior and treatment. State variability also suggests that the diversity of reported changes in gene expression levels in response to treatments such as fluoxetine may represent different perspectives on unified but noisy global gene expression system state level responses. Studying regulation of gene expression systems at the state level may be useful in guiding new approaches to augmentation of traditional antidepressant treatments.

## Introduction

Measurement of changes in gene expression levels in response to treatments is a commonly used approach to understanding biological processes. This is because gene expression levels frequently approximate protein levels, yet are much easier to measure. This is particularly true at the global level with gene expression profiling where the expression levels of nearly all genes can be measured in a single experiment. However, measurements of individual expression levels can also be problematic because of the sensitivity of these measurements to a multitude of factors. For instance differences in microarray platform and hybridization batch effects have often been blamed for difficulty in reproducing identified gene lists[1], [2]. Thus, there is a healthy skepticism about gene expression results and an expectation that results for individual genes will be confirmed with alternative methods. By contrast, we have found that the noisiness of gene expression measurements at the individual gene expression level does not translate to the systems level, where measurements of global gene expression system state, an aggregate measure of the behavior of thousands of gene expression levels such as those occurring during the progression of a developmental gene expression program, are highly robust[3], [4]. For instance, we, and others, have used covariance-based analyses such as principal components analysis (PCA), often referred to as singular value decomposition (SVD) when applied to gene expression data, to quantify the aggregate behavior of covarying gene expression levels[4], [5], [6], [7], [8], [9]. Such methods reduce thousands of gene expression measurement into principal components scores that describe the central tendency of large groups of covarying genes.

Because stereotyped gene expression programs, such as those occurring during development or in response to stimuli, are characterized by a large fraction of monotonically changing gene expression levels, we have found that the first principal component score (PCA1), which describes the monotonically changing fraction of genes, can be used to quantify the progression of gene expression programs under multiple conditions[4]. For instance, when principal components analysis (PCA) was performed on gene expression data from time course gene expression profiling experiments, such as during the development of neuronal subtypes or during the activation of T cells, the first principal component score (PCA1), a single measure for each microarray, changed monotonically across time[4]. Thus, PCA1 could arrange microarrays into their correct temporal order without temporal information. This indicated that PCA1 could be used as a quantitative measure of gene expression system states with respect to their sequential position along steterotyped gene expression programs. Because gene expression programs involve thousands of gene expression levels we have found that PCA1 as a measure of system state is very robust. In fact, PCA can be performed on any randomly chosen 2% of genes to give nearly identical values (Pearson r correlation coefficients >0.95) for PCA1 using independent groups of non-overlapping genes. Thus, PCA1 summarizes the behavior of thousands of covarying montonically changing gene expression levels into continuous quantitative measures that describe the aggregate state of gene expression systems as they progress along stereotyped gene expression programs.

System state measurements such as PCA1 are believed to be so robust because gene expression systems are hierarchical with multiple levels of cross-regulation[10]. Noise develops in hierarchical systems and can be transmitted from higher to lower levels, but importantly noise in expression levels is layered on top of biological information about the state of gene expression systems. Consequently, when analyzed in the aggregate, thousands of biologically noisy gene expression levels and their technically noisy measurements can nonetheless be reduced to extremely robust measures describing the overall state of a gene expression system[3], [4]. Measurement of gene expression system state is not only useful because it is robust, but also because these robust measures incorporate complex and often difficult to measure details of dynamic gene expression systems into single measures which can then be easily related to higher level processes such as cell function, animal behavior, or disease state. For example, variability across development in the global state of the gene expression system of fast-spiking interneurons (FS cells), as measured by PCA1, was related to developmental variability in FS cell function[4], [11]. Interestingly, we found that variability in the maturity of the FS cell gene expression system was not solely determined by the age of an individual[3]. Suggesting there was pathological importance to variability in the maturity of the FS cell program we found after controlling for age that the global state of the FS cell gene expression system was immature in the cortex of humans with autism, schizophrenia, and bipolar disorder[3]. Thus, system state measurements supported the hypothesis that immaturity of specific cell types in specific brain regions might contribute to neuropsychiatric disease.

Along similar lines we reasoned that using PCA1 to measure the global state of the gene expression system in the dentate gyrus, a brain region implicated in the pathogenesis of depression and the treatment response to antidepressant medication, might provide novel information about antidepressant treatment responses at the gene expression system level. Specifically we hypothesized that aggregate gene expression level responses to antidepressant treatment could be described at the system level and that variability in the state of the dentate gene expression system might explain observed variability in behavioral responses to treatment. Supporting these hypotheses, we describe a relationship between the global state of the dentate gyrus gene expression system and variability in antidepressant-sensitive behaviors in response to fluoxetine. Results indicate that state variability involves large-scale changes in thousands of genes, can be robustly measured with gene expression profiling combined with PCA, and can be used to relate variability in system state to higher order processes such as behavior and treatment response.

## Materials and Methods

### Ethics Statement

All animal work was conducted in compliance with the NIH laboratory animal care guidelines and with protocols approved by the Institutional Animal Care and Use Committee at Columbia University.

### Mice

Adult male C57BL/6Ntac mice were purchased from Taconic Farms (Germantown, NY, USA). All mice were 7–8 weeks old and weighed 23–35 g at the beginning of the treatment, were maintained on a 12L:12D schedule, and were housed five per cage. Food and water were provided ad libitum.

### Drugs

Treatments were carried out as previously described[12]. Corticosterone (CORT) (from Sigma, St. Louis, MO) was dissolved in vehicle (0.45% beta-cyclodextrin, Sigma, St Louis, MO). Fluoxetine hydrochloride (160 ug/ml) was purchased from Anawa Trading (Zurich, Switzerland). Corticosterone (35 ug/ml) was delivered alone or in the presence of antidepressant in opaque bottles to protect it from light, and was available ad libitum in the drinking water. In a separate cohort of mice serum levels of norfluoxetine, the active metabolite of fluoxetine, were comparable across mice treated with fluoxetine and CORT in the drinking water, and serum norfluoxetine levels were not related to behavior (Figure S3).

### Behavioral Testing

Behavioral tests were carried out as previously described[12]. The novelty suppressed feeding test (NSF) was done first, followed by the forced swim test (FST) 4 days later. The NSF test was carried out during an 8 min period as previously described[12]. Mice were exposed to twenty-four hours of food deprivation. Latency to eat a food pellet in the center of a brightly lit box was measured. For the FST, mice were placed into plastic buckets (19 cm diameter, 23 cm deep, filled with 23°C–25°C water) and videotaped for the entire 6-minute session. Immobility was considered to be when animals floated with no attempt at swimming.

### RNA extraction and microarray experiments

To allow for stress related to the FST to subside, mice were maintained on their drug regimen and left undisturbed one week following the end of behavioral experiments. Mice were then sacrificed and whole brains were dissected and placed into chilled ACSF solution for five minutes. The hippocampus was then dissected while maintaining the correct dorsal-ventral orientation. Transverse slices were cut through the hippocampus along the septotemporal axis and the molecular and granular layers of the dentate gyrus were microdissected from these transverse slices. Bilateral dentate gyri from dorsal or ventral sections of each mouse were combined into separate RNase free microcentrifuge tubes for each region. All samples were then immediately flash frozen and stored at −80 degrees Celsius. For RNA isolation, an appropriate volume of lysis buffer (Qiagen RNeasy kit) was added to the frozen tissue, which was then homogenized with a handheld tissue homogenizer. RNA was isolated following the manufacturer's instructions. Approximately 500 ng of high quality RNA was isolated per sample and prepared for a small scale Affymetrix protocol (requiring 100 ng). RNA was then submitted to Expression Analysis (expressionanalysis.com) for microarray processing. All samples were processed in parallel and hybridized in a single batch using Affymetrix 430_2 3′ expression arrays. Expression Analysis is Clinical Laboratory Improvement Amendments (CLIA) and Good Laboratory Practices (GLP) compliant and incorporates all CLIA and GLP quality control measures (QC) into its workflow (http://expressionanalysis.com/quality/quality_systems/).

### Microarray data processing

Affymetrix “.CEL” files were imported into Affymetrix Expression Console. Data processing for expression levels was performed with Robust Multiarray Analysis (RMA) and MicroArray Analysis Suite 5 (MAS5). Present calls were obtained from MAS5 analysis. Data were deposited in the Gene Expression Omnibus (GEO) accession number GSE43261.

### Principal components analysis (PCA)

**Figure 1:**
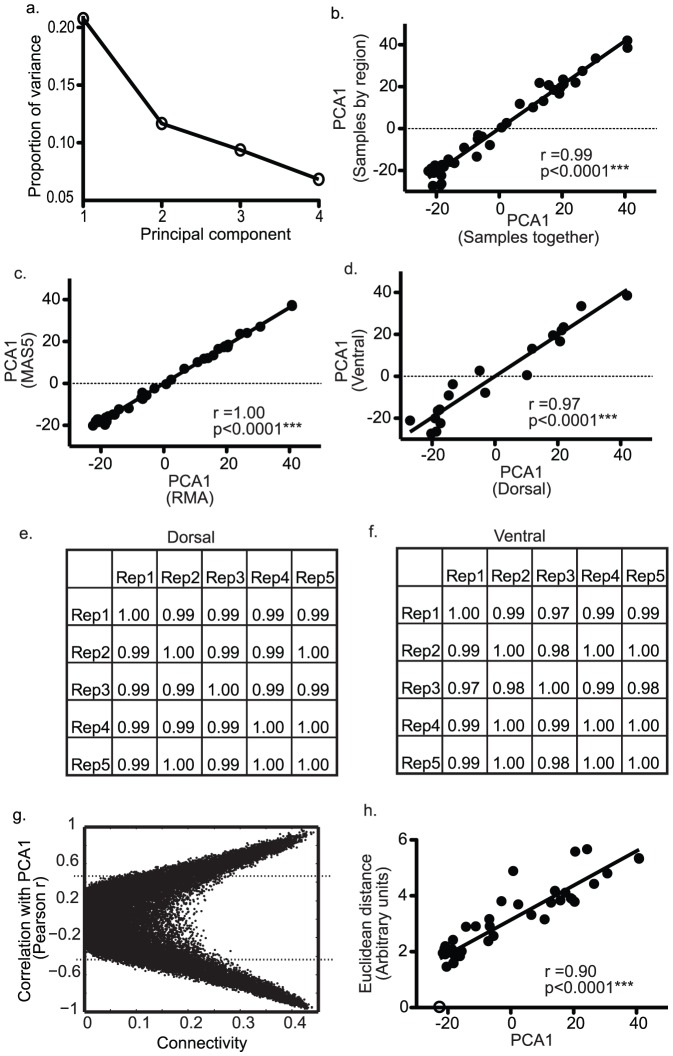
Principal components analysis generates a robust meaure (PCA1) of gene expression system state. Panel (a) plots the proportion of variance explained by principal components 1–4. Panel (b) is a bivariate plot of PCA1 values obtained from PCA done on all samples grouped together (x-axis) versus PCA1 from separate PCAs on samples grouped by region (y-axis) (r = 0.99, p<0.001). Panel (c) shows that the data processing algorithm used has no effect on values for PCA1 (r = 1.00, p<0.001). Panel (d) compares PCA1 values of dorsal and ventral dentate from the same mice (r = 0.97, p<0.001). Panels (e–f) are cross-correlation tables for PCA1 values obtained from PCA on independent groups of 2000 expression levels/group in dorsal (e) and ventral (f) dentate samples. Panel (g) plots gene connectivity (x-axis) against the correlation coefficient of genes with PCA1 demonstrating that more connected genes follow more closely with PCA1. Panel (h) plots PCA1 versus the Euclidean distance of transcriptomes from the transcriptome with the lowest value for PCA1 (open circle).

Five non-overlapping groups of expression levels from 2000 probe sets were each subjected to principal components analysis (PCA). Probe set groups were selected based on the order of their probe set ID numbers, i.e. Group 1 was probe sets #1-2000, Group 2 was probe sets #2001-4000, etc. PCA was performed in MATLAB on Z-scored data. Similar results could be obtained by randomly shuffling the probe sets before selecting them based on order, indicating that the order assigned by Affymetrix does not impact our results. The first principal component score (PCA1) was used as a measure of gene expression system state for each microarray. PCA1 from principal components analysis of each of the five groups of probe sets were nearly identical (Figure 1e–f).

### Correlations

Pearson correlations were used for data with normal distributions and Spearman correlations were used when data was not normally distributed.

### Statistics

Students' t-tests and correlation measures were calculated in Prism (GraphPad, La Jolla, CA).

### Connectivity analysis

An adjacency matrix was constructed from cross-correlations of gene expression levels. Genes were considered connected if they had a significant (p<0.001) cross-correlation. Connectivity was calculated as the number of connections/total number of genes.

### Euclidean distance

Euclidean distance was calculated in Matlab using the “pdist” function and measured relative to the transcriptome with the lowest value for PCA1.

### David Functional Annotation Clustering

Functional Annotation Clustering was done using the David Analysis Wizard (http://david.abcc.ncifcrf.gov/tools.jsp). Clustering analysis begins with a traditional pathway analysis in which a specified group of gene expression level identifiers is evaluated for pathway enrichment relative to background using a modified Fisher's exact test (EASE score). Clustering analysis then uses the redundancy of annotations to group pathways, with EASE scores below a cutoff, into biologically meaningful groups based on similarity of annotations. The Group Enrichment Score measures the geometric mean (in negative log10 scale) of the group member's p-values in the cluster. For example, an Enrichment Score of 5 would mean the average p-value for the pathways in a cluster was 10^-5^. Default setting modifications included: 1) Entering our own background expression levels, which was limited to genes that were defined as present by Affymetrix MAS5 analysis in greater than 50% of samples; 2) In order to reduce annotation redundancy, annotation databases were limited to one Functional Category, SP_PIR_KEYWORDS, and two ontology categories, GO_Biological Process and GO_Molecular Funtion; 3) To decrease the number of non-specific pathways used for clustering, the EASE cutoff was decreased to 0.05; and 4) To increase the clustering of related annotations, the similarity threshold was decreased to 0.35. All other values were left at their defaults. Finally, because there are often multiple probesets for each gene, probe sets were summarized into single expression values for each gene using a weighted average based on the proportion of present calls for a given probe set. Therefore, all gene lists entered into DAVID had single expressions values for each gene to prevent bias towards genes with multiple probe sets and to make sure genes were not duplicated across lists.

## Results

### Behavioral responses to fluoxetine (FLX) are variable in a mouse model of depression

Thirty mice were treated with chronic corticosterone (CORT), which induces depression-like behaviors with increased immobility in the Forced Swim Test (FST) and anxiety-like behavior with increased latency to eat in the Novelty Suppressed Feeding Test (NSF). These features can then be reversed with chronic antidepressant treatment[12], [13]. CORT was combined with vehicle (15 mice) or FLX (15 mice) for 21 days. Following treatment, mice were subjected to behavioral tests in the FST and NSF. At the group level, FLX significantly decreased immobility in the FST (p<0.005) and decreased latency to eat in the NSF (p<0.005) (Figure S1a–b). At the individual level, there was substantial inter-individual variability in the behavioral responses to fluoxetine. In general, immobility and latency were correlated (Figure S1c, Spearman r = 0.47, p = 0.008). However, a minority of mice (4 out of 15) appeared to respond to FLX in the NSF but not in the FST. Because we were interested in relating general variability in antidepressant-sensitive behaviors to global gene expression system level responses, behaviorally ambiguous mice were not used for microarray experiments described below, such that 11 out 15 fluoxetine treated mice were used for microarrays. In the future it may be interesting to evaluate ambiguous responders, as they could be useful in dissociating anti-depression-like effects from anti-anxiety-like effects. Eight randomly chosen mice not treated with FLX were used as representative controls for microarray studies.

### Similar gene expression responses to fluoxetine found in dorsal and ventral dentate gyrus

**Figure 2:**
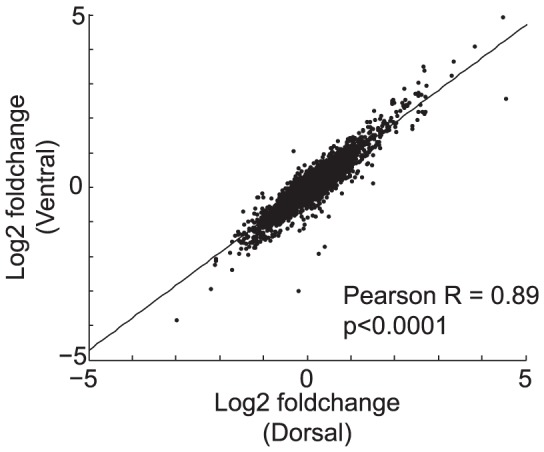
FLX-induced gene expression changes were the same in dorsal and ventral dentate samples. Gene expression profiling of the dorsal and ventral dentate gyrus of mice treated with FLX+CORT were compared to samples from mice treated with CORT only. Figure plots the log2-fold changes in gene expression levels from FLX+CORT samples relative to CORT only samples in dorsal (x-axis) versus ventral (y-axis) dentate gyrus. Log2 fold changes in gene expression levels were highly correlated (Pearson r = 0.89, p<0.0001) across regions.

Gene expression microarrays were used for whole transcriptome profiling of dentate gyrus tissue. Based on research demonstrating a distinction between dorsal (spatial) and ventral (emotional) hippocampal information processing[14], [15], [16], [17], [18], [19], the bilateral dentate gyri were separated into dorsal and ventral sections from each mouse. Log-fold changes in gene expression levels in response to FLX were compared between the dorsal and ventral dentate. To our surprise, responses were virtually identical across regions (Figure 2, Pearson R = 0.89, p<0.0001). The overall gene expression response involved thousands of genes with 46% of genes significantly changing expression levels in at least one region and 21% significantly changing in both regions. Supporting that the same response was occurring in both regions, 94% of genes that were significant in at least one region changed in the same direction in the other region, and 99.9% of genes that were significant in both regions changed in the same direction.

### Variability in gene expression levels in the dentate gyrus results from variability in the global state of the dentate gyrus gene expression system

Variability in gene expression levels can result from variability in the state of expression systems, which often involves stereotyped changes in thousands of genes, commonly referred to as gene expression programs[4], [20], [21]. We, and others, have found that covariance-based data analyses such as principal components analysis (PCA) can be used to study global changes in gene expression systems[4], [22], [23], [24], [25]. Though these methods are frequently used with time course data, variability in the state of gene expression systems, irrespective of the origin of variability, can be detected with PCA[4]. Previous work indicated that the first principal component score (PCA1) measures the global state of gene expression systems under many conditions[3], [4]. For example, PCA1 from developmental time course studies always increased monotonically over time, thus defining the global state of the system across development[4]. We, therefore, hypothesized that such an approach could quantify variability in the state of the dentate gene expression system in our experimental samples.

PCA was performed on gene expression data from multiple samples from FLX- treated and untreated mice that were grouped by region (dorsal or ventral dentate). PCA1 values, which explained approximately 20% of the variance in gene expression data (Figure 1a), for each sample were the same whether PCA was performed on all samples together or performed separately on samples grouped by region (Figure 1b, Pearson R = 0.99, p<0.0001). PCA1 values were also identical when microarray data were processed and summarized with RMA or MAS5 algorithms (Figure 1c, Pearson R = 0.99, p<0.0001). As was suggested by the dorsal/ventral regional comparisons above, PCA1 was the same across regions (Figure 1d, Pearson R = 0.97, p<0.0001). As previously reported, PCA was not affected by differences in the probe sets used for the principal components analysis. PCA performed on five independent groups of 2000 expression levels yielded nearly identical values for PCA1 (Figure 1e-f, R>0.97, p<0.001). The number of genes used was arbitrary and similar results were obtained with subsets of 500, 1000, and 4000 probe sets (not shown).

Principal components scores, sometimes referred to as Eigengenes[5], [7], [22], [26], describe the central tendency of gene expression levels that correlate with them, and therefore, represent an aggregate measure of large-scale stereotyped changes in gene expression systems. In this regard PCA1 was significantly positively correlated with the expression levels of greater than 6000 probe sets representing greater than 4000 unique genes and negatively correlated with nearly 7000 probe sets representing approximately 4,500 unique genes. As above with fluoxetine-induced gene expression changes, a high degree of overlap in expression levels that correlated with PCA1 was present between dorsal and ventral dentate samples. Thousands of expression levels, including 57% of expression levels detected as present, significantly correlated with PCA1 in at least one region and 28% were significantly correlated in both regions. Consistent with the hypothesis that genes were changing in the same way in both regions, 92% of expression levels that significantly correlated with PCA1 in at least one region correlated in the same direction in the other region, and 99.8% of expression levels that were significant in both regions were correlated in the same direction.

High levels of covariance occur as gene expression systems vary because these systems have hierarchical structures [10], [27], [28], [29]. Thus, in the current experiment the connectivity of genes, a measure of a gene's position in a hierarchy with highly connected genes at the top of hierarchical systems, was related to the degree to which genes followed PCA1. Regardless of the direction of the correlation, more connected genes followed more closely with PCA1 (Figure 1g). Further demonstrating that PCA1 measured global changes in system state, the Euclidean distance, a global measure of dissimilarity, from the transcriptome with the lowest value for PCA1 was linearly related to PCA1 (Figure 1h).

### Levels of antidepressant-sensitive behaviors relate to the global state of the dentate gene expression system

**Figure 3:**
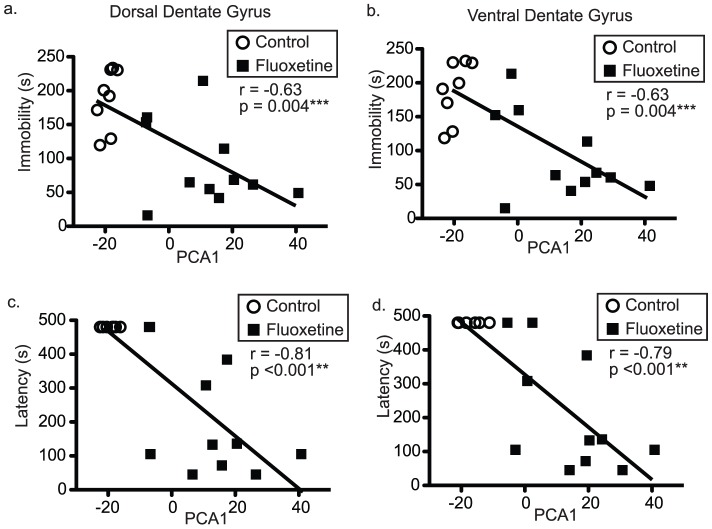
PCA1 is inversely related to levels of antidepressant-sensitive behaviors. Panels (a–b) plot immobility in the FST (y-axis) against PCA1 (x-axis) for the dorsal (a, Spearman r = −0.63, p = 0.004) and ventral (b, Spearman r = −0.63, p = 0.004) dentate gyrus samples. Panels (c–d) plot latency to eat in the NSF (y-axis) against PCA1 (x-axis) for the dorsal (c, Spearman r = −0.81, p = 0.004) and ventral (d, Spearman r = −0.79, p = 0.004) dentate gyrus samples.

Because fluoxetine-induced gene expression changes and PCA1-correlated expression levels both included greater than 50% of all present genes and were similar across dorsal and ventral dentate, it was predicted that genes induced by fluoxetine would be the same as those following PCA1. In fact, 90% of gene expression levels significantly altered by fluoxetine in at least one region of the dentate were also significantly correlated with PCA1 in at least one region. In all cases, genes that were significantly positively correlated with PCA1 were upregulated by fluoxetine and vice versa. Thus, PCA1 measured aggregate changes in thousands of genes responding to fluoxetine and suggested PCA1 could be used to relate variability in dentate gyrus gene expression system state to variability in antidepressant-sensitive behaviors in response to fluoxetine. Regression analyses indicated that for both dentate regions and for both behavioral measures there were highly significant correlations between system state (PCA1) and behavior (Figure 3: Immobility in the FST – ventral: Spearman r = −0.63, p = 0.004***, dorsal: Spearman r = −0.63, p = 0.004***; Latency to eat in the NSF – ventral: Spearman r = −0.79, p<0.001***, dorsal: Spearman r = −0.81, p<0.001***).

### Pathway analysis of PCA-identified genes confirms measurement of a biological signal

Much concern exists about gene expression profiling studies, particularly microarray studies, and a potential for type 1 errors and/or technically related gene expression changes being mistaken for biologically related changes. With traditional gene expression approaches these concerns are addressed with the use of alternative quantitative measures such as real-time PCR or protein level quantification. However, our measure, PCA1, captures the behavior of thousands of genes. Validating any number of these genes still would not validate that PCA1 truly measured a biologically meaningful change in the dentate gene expression system. To address this question, we used Functional Annotation Clustering to demonstrate that PCA1-related genes fell into biologically cohesive groups, whereas, PCA1-unrelated genes did not, or at least to a much lesser degree. Enriched annotation pathways were identified using pathway analysis with the David Functional Annotation Tool and clustered by similarity into groups with shared biological themes. Enrichment Scores, which are negative log10 measures of the average significance of multiple related annotation pathways in a cluster, were used to quantify the significance of the biological annotation clusters (see Methods). A large cluster of 26 annotation pathways with an enrichment score of 6.0 was identified for significantly positively PCA1-correlated genes and was centered on the biological theme of membrane signaling (Table S1). Interestingly, BDNF, which was highly correlated with PCA1 (Figure S2: r = 0.93, P<0.001) and has been widely reported to be an important player in antidepressant treatment responses[30], [31], [32], [33], was identified in this cluster. The remaining two out of the top three clusters for this group of genes all had enrichment scores greater than 3.8. For the significantly negatively correlated group of genes the top Enrichment Score of 3.45 was for a cluster of annotation pathways centered on the biological theme of ribosomes and translation (Table S2). For non-significantly correlated genes the highest Enrichment Score for either group was 2.1, indicating that p-values for enriched pathways in groups of genes related to PCA1 were much more significant than pathways in groups of PCA1-unrelated genes. To further support the validity of identified clusters, groups of genes were randomly split in half and the split groups of genes were analyzed separately. Two out of the top three clusters with the highest enrichment scores for both significantly positively and negatively correlated genes demonstrated the same cluster themes and many of the same pathways in the split groups of genes even though these groups did not share any of the same genes (Table S3). On the other hand, analysis of subgroups from non-significant genes did not identify any shared clusters or shared pathways. Of note, a list of all probe sets that were significantly changed in response to fluoxetine in both the dorsal and ventral dentate (Table S4) is provided for researchers who wish to compare gene lists. Additionally, raw.CEL files have been deposited in the Gene Expression Omnibus (GEO) accession number GSE43261.

## Discussion

Principal components analysis, also known as singular value decomposition (SVD), has been widely used to analyze gene expression data[5], [9]. Historically these techniques have been used to identify and classify groups of genes that behave in a coherent manner. In this regard, much emphasis has been placed on validating biological covariance. For instance, techniques such as weighted gene network connectivity analysis (WGNCA)[22], [23] and gene expression clustering methods[28], start with PCA or similar methods, but then refine groups of covarying genes by using dissimilarity measures to further divide groups of covarying genes into subgroups of genes with even more similar dynamics. WGNCA goes even a step further by refining gene networks using known biological interactions of gene-encoded proteins to give added weight to expression level relationships that have support at the protein interaction level. Thus, the focus of these techniques is to define, with as much confidence as possible, groups of genes that biologically covary to better understand the nature of the responses being studied. By contrast, we focus on PCA1 itself as a measure of an overall gene expression system state and go to great lengths to show that the same values for PCA1 were obtained no matter what genes (any random 2% of genes), data summarization method (MAS5 vs. RMA), or sample grouping (by region or all together) was used. Thus, while great care must be taken when the goal is to accurately determine which individual genes are truly parts of a co-regulatory group, it is paradoxically very difficult not to get the same values for system state measures no matter what method is used. We interpret this to mean that there is a higher level of organization for co-regulatory networks at the system state level and that PCA1 quantifies this higher organizational state. Quantifying system states demonstrates the continuity of state transitions, lends itself to robustly quantifying the extent of global system level changes, and helps relate system state changes to higher order processes such as disease, behavior, and treatment response.

We feel system state measures are particularly important because they measure an aspect of gene expression system regulation that could potentially be exploited for treatment benefit. While it is unclear in the current study why certain individuals responded differently to antidepressant treatment, the relationship between treatment, behavior, and systemic transcriptional responses in a brain region implicated in antidepressant-sensitive behaviors strongly suggests that the observed gene expression response has some functional relevance to treatment effects. Importantly, if gene expression changes are causally related to a treatment responses and if gene expression changes occur as part of large-scale changes in gene expression systems then understanding how to augment therapeutically beneficial changes in gene expression systems becomes an important research goal. Though somewhat counterintuitive and paradoxical, the more important an aggregate systemic gene expression response is to observed treatment effects, the less important to treatment strategies it may be to understand the details of the response. For instance, large-scale modulation of epigenetic regulators, such as with histone deacetylase inhbitors (HDACi), is often associated with the enhancement of multiple biological responses from memory formation[34], [35], [36] to immune responses[37], [38], [39], [40] to cancer therapy [41], [42], [43]. Using HDACi as an example, gene expression system state measures provide a method to determine whether HDACi act by modulating endogenous gene expression system responses for therapeutic benefit. If this is the case, then an important goal becomes how to target these system-modulating therapies to brain regions where global chromatin modification would be beneficial, while avoiding areas where chromatin restructuring would be unnecessary or potentially harmful. Such a localizing strategy is well established within oncology, which may be a useful source for future guidance. In other words, if a response in its entirety can be modulated, measured, and can elicit therapeutic effects, then understanding the details of the response, while potentially interesting, is not necessary to develop rational treatment strategies. It is only when an aggregate gene expression system level response does not explain a biological response or cannot be targeted or manipulated for therapeutic benefit that system details offer a potential therapeutic work-around. While it is tempting to suggest that treatments targeted to limited aspects of system-wide responses may have lower risk of side-effects, it can alternatively be argued that mimicking healthy large-scale biological responses may be the most naturalistic and effective therapeutic strategy. System state measures provide a useful tool to evaluate these contrasting perspectives.

It is also important to discuss our approach to experimental validation, which was different from traditional gene expression studies and did not involve real-time PCR or protein quantification methods. These methods are typically used to validate changes in specific genes to define which gene expression changes merit an investment of time and resources for further study. Validating a systemic measure, however, cannot be accomplished at the level of individual genes and required different approaches to support results. One validation approach we used was subsampling of independent groups of genes to show that PCA1 values obtained using any subsample of genes were the same as PCA1 values from other completely independent subsets of genes. Subsampling demonstrates that PCA1 measures a systemic property that is widely distributed across the transcriptome. Similarly we showed that the choice of microarray data processing algorithms did not affect results even though processing algorithms are known to give different results at the level of individual genes and in the reverse engineering of gene expression networks[44]. Thus, redundant information about the state of the gene expression systems was able to overcome potential effects of variability in individual gene expression levels introduced by processing algorithms. Another way we supported our results was to demonstrate that genes that correlated with PCA1 were enriched for biological pathways relative to PCA1-unrelated genes. This approach indicated that PCA1 was unlikely to be measuring a technical artifact, which should not enrich for biological pathways. As discussed above, the importance of measuring gene expression system state is not to highlight details of the response, thus, the purpose of pathway analysis in this study was not to focus on the identified pathways, but rather to address potential concerns that we were measuring a technical artifact. The finding that PCA1-related genes were predominantly the same genes that were induced by fluoxetine demonstrated in another way that PCA1-related genes were in the aggregate part of a biological response. Finally, the relationship of PCA1 to two antidepressant-sensitive behavioral measures added another level of support to the idea that gene expression system states might contribute to behavioral effects, which suggests future experiments designed to understand whether system states could be modulated for therapeutic benefit.

Comparing gene expression measures across dorsal and ventral dentate was also interesting. There was a high level of covariance across these spatially, and reportedly functionally, distinct subregions [14], [15], [16], [17], [18], [19], [45], [46]. This included covariance in system state measures and individual gene expression levels. In the current experiment all mice were exposed to systemic treatments (CORT or CORT +FLX), therefore, it is possible that state covariance across regions only occurs in the presence of systemic signals. Thus, it would be interesting to study whether regional covariance of system state exists under more naturalistic conditions. Nonetheless, the current experiment demonstrates that at least with systemic exposures such as treatments, dentate regions can be synchronized at the gene expression system state level. Thus, if antidepressant treatments are able to impact different aspects of cognitive processing that are subserved by the dorsal and ventral dentate, these effects are likely the consequence of the same gene expression level changes having different effects based on cellular location and circuit level integration. Another consideration with respect to our systemic treatments is their delivery via drinking water. Separate studies documented that comparable serum levels of norfluoxetine, the active metabolite of fluoxetine, were reached in all CORT + FLX-treated mice and that there was not a relationship between norfluoxetine levels and behavioral responses (Figure S3). Importantly, the goal of the current study was not to determine which factors, e.g. dosing, epigenetic differences, etc. contributed to variability in antidepressant-sensitive behaviors. The variability, however it was generated, was used to demonstrate covariance of systemic gene expression measures with behavior. Follow up studies might include determining whether variability in behavioral responses induced by different factors such as variable dosing [47], genetic factors[48], [49], [50], or epigenetic factors [51], [52], [53], [54] would demonstrate the same relationship between dentate system state, treatment, and behavior. Such a convergent relationship between dentate system state and treatment response variability would help prioritize gene expression system state as a promising treatment target. Similarly, it would be interesting to determine whether gene expression system state measures in other brain regions also covary with the current or other measures of behavior.

Another interesting question raised by our study is in what cell type or types are gene expression systems changing? A relationship between gene connectivity and gene expression system state supports a hierarchical organization to system level changes, however, a hierarchical data structure could be the product of intra or inter-cell type signaling cascades or both. Fluoxetine has been reported to have diverse effects in multiple cell types and brain regions[31], [32]. Therefore, cell type specific purification techniques such as laser-capture or bacTRAP purification[49] would be useful for future study of this interesting question.

In summary, our study applied a novel approach to gene expression systems research to show that a gene expression system-wide response in the dentate gyrus to fluoxetine involving thousands of expression levels could be captured in a single robust measure (PCA1) with PCA, which was significantly related to variability in antidepressant-sensitive behaviors. These results and our state measurement approach set the stage for future efforts to determine mechanisms by which gene expression system state is modulated by fluoxetine and other antidepressants. Such experiments may include dentate-specific genetic [48], [49], [50] and/or epigenetic [51], [52], [53], [54] manipulations, which could help establish a causal relationship of system state modulation to treatment response variability and point the direction towards novel augmentation strategies for individuals resistant to current antidepressant treatments.

## Supporting Information

Figure S1 **Variability present in behavioral response to fluoxetine.** All mice (n = 30) were chronically (21 days) treated with corticosterone (CORT) (35 ug/ml) in the drinking water. Fifteen mice were also co-administered fluoxetine (FLX) (160 ug/ml). Following chronic treatment mice were tested in the Forced Swim Test (FST) and Novelty Suppressed Feeding paradigm (NSF). At the group level FLX-treated mice demonstrated significantly decreased latency to eat in the NSF (panel a: P<0.005) and decreased immobility in the NSF (panel b: P<0.005). At the individual level there was a significantly correlation between latency to eat (x-axis) and immobility (y-axis) (Spearman r = 0.47, p = 0.008). Four mice (Ambiguous – open triangles) appeared to respond in the NSF but not in the FST. These mice were not used for microarray experiments.
(DOCX)

Figure S2 **BDNF expression levels were highly correlated with PCA1.** Two probe sets for BDNF were present on the Affymetrix microarray platform used in the current study. Expression levels for these probe sets were highly correlated (panel a, Spearman r = 0.99, p<0.0001). A single BDNF level for the two probe sets was calculated as the average of log2 transformed mean-standardized expression levels. Average BDNF levels were highly correlated with PCA1 (panel b, Spearmen r = 0.93, p<0.0001).
(DOCX)

Figure S3 Serum norfluoxetine levels are similar across mice that receive the same concentration of fluoxetine in drinking water and are not related to levels of antidepressant sensitive behaviors. A separate cohort of eighteen mice was treated for 21 days with fluoxetine (160 ug/ml) in the drinking water. Serum norfluoxetine levels were measured at the time of sacrifice. Panel (a) shows that behavioral responses were variable and that latency to eat in the NSF was correlated with immobility in the FST (Spearman r = 0.64, p = 0.005). Panels (b–c) show that there were comparable serum levels (575–725 ng/ml) of norfluoxetine in all mice and that variability in levels was not related to behavioral measures.
(DOCX)

Table S1 **Annotation Cluster analysis of PCA1-related genes reveals a biological signal (positively correlated genes).** Functional Annotation Clustering was done on lists of genes that were significantly positively correlated with PCA1. Results were compared to Functional Annotation Clustering on lists of genes that were non- significantly positively correlated with PCA1. Gene lists were culled to identical sizes based on the random removal of genes to make all lists contain 1,600 genes. Gene lists were also split into random subgroups of 800 genes each for independent analyses. When multiple probe sets were present for genes, results were summarized to a single value based on a weighted average with weights assigned by the percentage of present calls across all samples. Table S1 shows the top 3 annotation clusters for significantly positively correlated genes (left) and non- significantly positively correlated genes (right).
(DOCX)

Table S2 **Annotation Cluster analysis of PCA1-related genes reveals a biological signal (negatively correlated genes).** Functional Annotation Clustering was done on lists of genes that were significantly negatively correlated with PCA1. Results were compared to Functional Annotation Clustering on lists of genes that were non- significantly negatively correlated with PCA1. Gene lists were culled to identical sizes based on the random removal of genes to make all lists contain 1,600 genes. Gene lists were also split into random subgroups of 800 genes each for independent analyses. When multiple probe sets were present for genes, results were summarized to a single value based on a weighted average with weights assigned by the percentage of present calls across all samples. Table S2 shows the top 3 annotation clusters for significantly negatively correlated genes (left) and non- significantly negatively correlated genes (right).
(DOCX)

Tables S3 **Annotation Cluster analysis of PCA1-related genes reveals a biological signal (randomly split gene lists).** Functional Annotation Clustering was done on lists of genes that were significantly positively or negatively correlated with PCA1. Results were compared to Functional Annotation Clustering on lists of genes that were non- significantly positively or negatively correlated with PCA1. Gene lists were culled to identical sizes based on the random removal of genes to make all lists contain 1,600 genes. Gene lists were also split into random subgroups of 800 genes each for independent analyses. When multiple probe sets were present for genes, results were summarized to a single value based on a weighted average with weights assigned by the percentage of present calls across all samples. Table S3 shows the similarity of annotation clusters and pathways from randomly split lists (800 non-overlapping genes) of significantly positively and negatively PCA1-correlated genes. Shared pathways are highlighted in bold.
(DOCX)

Table S4 List of probe sets that were signicantly up- or down-regulated in both the dorsal and ventral dentate in response to fluoxetine. Lists are arranged in descending order according to absolute average log-fold change across regions.
(DOCX)
