# Supplementary material for: Global State Measures of the Dentate Gyrus Gene Expression System Predict Antidepressant-Sensitive Behaviors
Source: PLoS One. 2014 Jan 17;9(1):e85136. doi: 10.1371/journal.pone.0085136 (PMC3894967; doi:10.1371/journal.pone.0085136)
Supplement: Figure S1 — Variability present in behavioral response to fluoxetine. All mice (n = 30) were chronically (21 days) treated with corticosterone (CORT) (35 ug/ml) in the drinking water. Fifteen mice were also co-administered fluoxetine (FLX) (160 ug/ml). Following chronic treatment mice were tested in the Forced Swim Test (FST) and Novelty Suppressed Feeding paradigm (NSF). At the group level FLX-treated mice demonstrated significantly decreased latency to eat in the NSF (panel a: P<0.005) and decreased immobility in the NSF (panel b: P<0.005). At the individual level there was a significantly correlation between latency to eat (x-axis) and immobility (y-axis) (Spearman r = 0.47, p = 0.008). Four mice (Ambiguous – open triangles) appeared to respond in the NSF but not in the FST. These mice were not used for microarray experiments. (DOCX) [file pone.0085136.s001.docx]

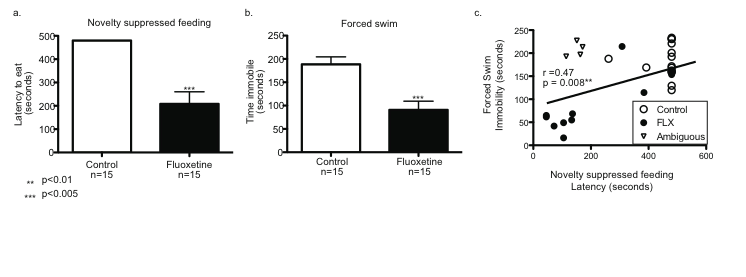


**Figure S1: Variability present in behavioral response to fluoxetine.** All mice (n = 30) were chronically (21 days) treated with corticosterone (CORT) (35 ug/ml) in the drinking water. Fifteen mice were also co-administered fluoxetine (FLX) (160ug/ml). Following chronic treatment mice were tested in the Forced Swim Test (FST) and Novelty Suppressed Feeding paradigm (NSF). At the group level FLX-treated mice demonstrated significantly decreased latency to eat in the NSF (panel a: P < 0.005) and decreased immobility in the NSF (panel b: P < 0.005). At the individual level there was a significantly correlation between latency to eat (x-axis) and immobility (y-axis) (Spearman r = 0.47, p = 0.008). Four mice (Ambiguous – open triangles) appeared to respond in the NSF but not in the FST. These mice were not used for microarray experiments.
