# Supplementary material for: Global State Measures of the Dentate Gyrus Gene Expression System Predict Antidepressant-Sensitive Behaviors
Source: PLoS One. 2014 Jan 17;9(1):e85136. doi: 10.1371/journal.pone.0085136 (PMC3894967; doi:10.1371/journal.pone.0085136)
Supplement: Figure S2 — BDNF expression levels were highly correlated with PCA1. Two probe sets for BDNF were present on the Affymetrix microarray platform used in the current study. Expression levels for these probe sets were highly correlated (panel a, Spearman r = 0.99, p<0.0001). A single BDNF level for the two probe sets was calculated as the average of log2 transformed mean-standardized expression levels. Average BDNF levels were highly correlated with PCA1 (panel b, Spearmen r = 0.93, p<0.0001). (DOCX) [file pone.0085136.s002.docx]

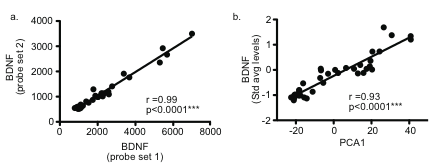


**Figure S2: BDNF expression levels were highly correlated with PCA1.** Two probe sets for BDNF were present on the Affymetrix microarray platform used in the current study. Expression levels for these probe sets were highly correlated (panel a, Spearman r = 0.99, p < 0.0001). A single BDNF level for the two probe sets was calculated as the average of log2 transformed mean-standardized expression levels. Average BDNF levels were highly correlated with PCA1 (panel b, Spearmen r=0.93, p < 0.0001).
