# Supplementary material for: Global State Measures of the Dentate Gyrus Gene Expression System Predict Antidepressant-Sensitive Behaviors
Source: PLoS One. 2014 Jan 17;9(1):e85136. doi: 10.1371/journal.pone.0085136 (PMC3894967; doi:10.1371/journal.pone.0085136)
Supplement: Figure S3 — Serum norfluoxetine levels are similar across mice that receive the same concentration of fluoxetine in drinking water and are not related to levels of antidepressant sensitive behaviors. A separate cohort of eighteen mice was treated for 21 days with fluoxetine (160 ug/ml) in the drinking water. Serum norfluoxetine levels were measured at the time of sacrifice. Panel (a) shows that behavioral responses were variable and that latency to eat in the NSF was correlated with immobility in the FST (Spearman r = 0.64, p = 0.005). Panels (b–c) show that there were comparable serum levels (575–725 ng/ml) of norfluoxetine in all mice and that variability in levels was not related to behavioral measures. (DOCX) [file pone.0085136.s003.docx]

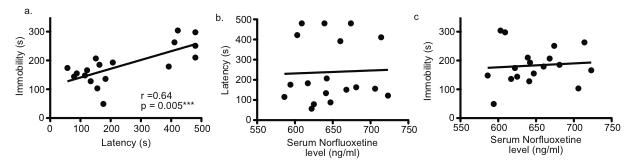


**Figure S3: Serum norfluoxetine levels are similar across mice that receive the same concentration of fluoxetine in drinking water and are not related to levels of antidepressant sensitive behaviors.** A separate cohort of eighteen mice was treated for 21 days with fluoxetine (160ug/ml) corticosterone (35 ug/ml) in the drinking water. Serum norfluoxetine levels were measured at the time of sacrifice. Panel (a) shows that behavioral responses were variable and that latency to eat in the NSF was correlated with immobility in the FST (Spearman r = 0.64, p = 0.005). Panels (b-c) show that there were comparable serum levels (575-725ng/ml) of norfluoxetine in all mice and that variability in levels was not related to behavioral measures.
