# Supplementary material for: Global State Measures of the Dentate Gyrus Gene Expression System Predict Antidepressant-Sensitive Behaviors
Source: PLoS One. 2014 Jan 17;9(1):e85136. doi: 10.1371/journal.pone.0085136 (PMC3894967; doi:10.1371/journal.pone.0085136)
Supplement: Tables S3 — Annotation Cluster analysis of PCA1-related genes reveals a biological signal (randomly split gene lists). Functional Annotation Clustering was done on lists of genes that were significantly positively or negatively correlated with PCA1. Results were compared to Functional Annotation Clustering on lists of genes that were non- significantly positively or negatively correlated with PCA1. Gene lists were culled to identical sizes based on the random removal of genes to make all lists contain 1,600 genes. Gene lists were also split into random subgroups of 800 genes each for independent analyses. When multiple probe sets were present for genes, results were summarized to a single value based on a weighted average with weights assigned by the percentage of present calls across all samples. Table S3 shows the similarity of annotation clusters and pathways from randomly split lists (800 non-overlapping genes) of significantly positively and negatively PCA1-correlated genes. Shared pathways are highlighted in bold. (DOCX) [file pone.0085136.s006.docx]

**
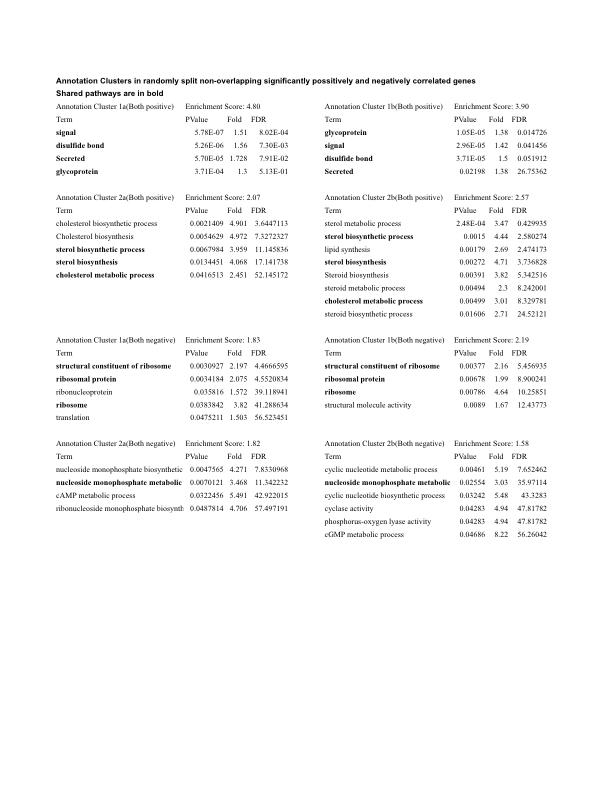
**

**Table S3: Annotation Cluster analysis of PCA1-related genes reveals a biological signal.** Functional Annotation Clustering was done on lists of genes that were significantly positively or negatively correlated with PCA1. Results were compared to Functional Annotation Clustering on lists of genes that were non- significantly positively or negatively correlated with PCA1. Gene lists were culled to identical sizes based on the random removal of genes to make all lists contain 1,600 genes. Gene lists were also split into random subgroups of 800 genes each for independent analyses. When multiple probe sets were present for genes, results were summarized to a single value based on a weighted average with weights assigned by the percentage of present calls across all samples. Table 3 shows the similarity of annotation clusters and pathways from randomly split lists (800 non-overlapping genes) of significantly positively and negatively PCA1-correlated genes. Shared pathways are highlighted in bold.
