# Supplementary material for: Global State Measures of the Dentate Gyrus Gene Expression System Predict Antidepressant-Sensitive Behaviors
Source: PLoS One. 2014 Jan 17;9(1):e85136. doi: 10.1371/journal.pone.0085136 (PMC3894967; doi:10.1371/journal.pone.0085136)
Supplement: Table S4 — List of probe sets that were signicantly up- or down-regulated in both the dorsal and ventral dentate in response to fluoxetine. Lists are arranged in descending order according to absolute average log-fold change across regions. (DOCX) [file pone.0085136.s007.docx]

Table S4: List of probe sets that were signicantly up- or down-regulated in both the dorsal and ventral dentate in response to fluoxetine. Lists are arranged in descending order according to absolute average log-fold change across regions.

| Upregulated in response to fluoxetine | | |  | Downregulated in response to fluoxetine | | |
| --- | --- | --- | --- | --- | --- | --- |
| ID | SYMBOL | AVG_LOG-FOLD |  | ID | SYMBOL | AVG_LOG-FOLD |
| 1424525_at | Grp | 3.90 |  | 1435500_at | Rab26 | -2.64 |
| 1427038_at | Penk | 3.36 |  | 1421595_at | Fam184b | -2.49 |
| 1442226_at | Sema3e | 3.16 |  | 1434802_s_at | Ntf3 | -2.36 |
| 1448673_at | Pvrl3 | 3.13 |  | 1454768_at | Kcnf1 | -2.35 |
| 1420720_at | Nptx2 | 3.08 |  | 1416658_at | Frzb | -2.05 |
| 1445642_at | Lemd1 | 2.92 |  | 1457702_at | Gpr12 | -1.92 |
| 1459269_at | Sdr39u1 | 2.76 |  | 1419093_at | Tdo2 | -1.87 |
| 1448556_at | Prlr | 2.74 |  | 1437371_at | Fam160a1 | -1.86 |
| 1442845_at | C130075A20Rik | 2.73 |  | 1442174_at | Tspan18 | -1.85 |
| 1448285_at | Rgs4 | 2.67 |  | 1433652_at | Igsf1 | -1.81 |
| 1417262_at | Ptgs2 | 2.66 |  | 1441509_at | A130009I22Rik | -1.80 |
| 1431946_a_at | Necab3 | 2.62 |  | 1418304_at | Cdhr1 | -1.77 |
| 1423407_a_at | Fbln2 | 2.57 |  | 1434785_at | Cacng5 | -1.76 |
| 1449151_at | Cdk18 | 2.54 |  | 1460203_at | Itpr1 | -1.75 |
| 1439569_at | Gpr83 | 2.50 |  | 1433681_x_at | Capn3 | -1.75 |
| 1456051_at | Drd1a | 2.41 |  | 1439086_at | A930009L07Rik | -1.75 |
| 1422053_at | Inhba | 2.34 |  | 1457729_at | --- | -1.71 |
| 1423365_at | Cacna1g | 2.27 |  | 1452114_s_at | Igfbp5 | -1.67 |
| 1433776_at | Lhfp | 2.27 |  | 1455686_at | Lcorl | -1.64 |
| 1448594_at | Wisp1 | 2.11 |  | 1456934_at | Calb1 | -1.63 |
| 1436662_at | Sorcs1 | 2.10 |  | 1422711_a_at | Pnck | -1.62 |
| 1420416_at | Sema3a | 2.07 |  | 1418104_at | Nrip3 | -1.61 |
| 1437247_at | Fosl2 | 2.05 |  | 1433716_x_at | Gfra2 | -1.58 |
| 1419411_at | Tac2 | 2.00 |  | 1450448_at | Stc1 | -1.54 |
| 1426508_at | Gfap | 1.96 |  | 1460482_at | 3110047P20Rik | -1.50 |
| 1449109_at | Socs2 | 1.92 |  | 1424007_at | Gdf10 | -1.48 |
| 1443225_at | Acvr1c | 1.90 |  | 1451342_at | Spon1 | -1.48 |
| 1440484_at | Unc5d | 1.86 |  | 1428397_at | B3galt5 | -1.46 |
| 1431749_a_at | Rasgrp1 | 1.77 |  | 1422643_at | Moxd1 | -1.45 |
| 1427883_a_at | Col3a1 | 1.75 |  | 1453321_at | Fndc1 | -1.45 |
| 1429549_at | Col27a1 | 1.74 |  | 1452366_at | Csgalnact1 | -1.43 |
| 1422168_a_at | Bdnf | 1.71 |  | 1460574_at | Fat4 | -1.43 |
| 1418021_at | C4b | 1.71 |  | 1436948_a_at | Fam70a | -1.43 |
| 1422437_at | Col5a2 | 1.66 |  | 1429897_a_at | D16Ertd472e | -1.43 |
| 1422706_at | Pmepa1 | 1.64 |  | 1456318_at | Clec1a | -1.41 |
| 1436094_at | Vgf | 1.62 |  | 1427306_at | Ryr1 | -1.39 |
| 1428549_at | Ccdc3 | 1.61 |  | 1438540_at | Col25a1 | -1.37 |
| 1416168_at | Serpinf1 | 1.56 |  | 1448823_at | Cxcl12 | -1.36 |
| 1441790_at | --- | 1.56 |  | 1419442_at | Matn2 | -1.35 |
| 1453125_at | Sox11 | 1.53 |  | 1421818_at | Bcl6 | -1.35 |
| 1428118_at | Lingo1 | 1.52 |  | 1421834_at | Pip5k1b | -1.35 |
| 1449188_at | Midn | 1.52 |  | 1437385_at | Ccbe1 | -1.34 |
| 1455869_at | --- | 1.51 |  | 1418393_a_at | Itga7 | -1.34 |
| 1441823_at | Zmiz1 | 1.49 |  | 1418671_at | Capn5 | -1.34 |
| 1434719_at | A2m | 1.49 |  | 1434742_s_at | Aifm3 | -1.32 |
| 1434376_at | Cd44 | 1.49 |  | 1455750_at | Ralgapa2 | -1.29 |
| 1416405_at | Bgn | 1.47 |  | 1429443_at | Cpne4 | -1.25 |
| 1449283_a_at | Mapk12 | 1.47 |  | 1417391_a_at | Il16 | -1.23 |
| 1441746_at | --- | 1.45 |  | 1454656_at | Spata13 | -1.22 |
| 1449131_s_at | Cd1d1 | 1.44 |  | 1425788_a_at | Echdc2 | -1.22 |
| 1416132_at | Efr3a | 1.43 |  | 1439843_at | Camk4 | -1.22 |
| 1421375_a_at | S100a6 | 1.41 |  | 1442263_at | Rgs13 | -1.21 |
| 1451280_at | Arpp21 | 1.40 |  | 1428851_at | 1300014I06Rik | -1.18 |
| 1429929_at | Mei1 | 1.40 |  | 1434736_at | Hlf | -1.17 |
| 1418149_at | Chga | 1.39 |  | 1439854_at | Hrk | -1.17 |
| 1419184_a_at | Fhl2 | 1.37 |  | 1437064_at | Ar | -1.17 |
| 1451406_a_at | Pcsk5 | 1.35 |  | 1435749_at | Gda | -1.16 |
| 1431225_at | --- | 1.34 |  | 1417673_at | Grb14 | -1.15 |
| 1423947_at | 1110008P14Rik | 1.33 |  | 1448812_at | Hpcal1 | -1.15 |
| 1427005_at | Plk2 | 1.32 |  | 1455760_at | Slc9a5 | -1.14 |
| 1449623_at | Txnrd3 | 1.31 |  | 1449980_a_at | Gabrd | -1.14 |
| 1431213_a_at | Gm3579 | 1.30 |  | 1435694_at | Arhgap26 | -1.14 |
| 1449519_at | Gadd45a | 1.30 |  | 1457440_at | Sstr4 | -1.13 |
| 1427229_at | Hmgcr | 1.28 |  | 1429123_at | Rab27a | -1.13 |
| 1455179_at | Mpp7 | 1.27 |  | 1455765_a_at | Abcc8 | -1.12 |
| 1452652_at | Tmem158 | 1.25 |  | 1439899_at | Galnt13 | -1.12 |
| 1453365_at | Rabgap1l | 1.23 |  | 1423630_at | Cygb | -1.12 |
| 1418674_at | Osmr | 1.23 |  | 1440849_at | Rasgrf2 | -1.12 |
| 1438864_at | --- | 1.22 |  | 1427501_at | 4922501L14Rik | -1.11 |
| 1416379_at | Panx1 | 1.22 |  | 1421943_at | Tgfa | -1.11 |
| 1435458_at | Pim1 | 1.22 |  | 1427049_s_at | Smo | -1.10 |
| 1460009_at | Ier5 | 1.21 |  | 1435848_at | D430041D05Rik | -1.10 |
| 1423566_a_at | Hsph1 | 1.21 |  | 1433639_at | Fam117a | -1.10 |
| 1417406_at | Sertad1 | 1.21 |  | 1425257_at | Acot5 | -1.09 |
| 1450754_at | Cacna2d2 | 1.18 |  | 1450798_at | Tnxb | -1.08 |
| 1422256_at | Sstr2 | 1.18 |  | 1418094_s_at | Car4 | -1.08 |
| 1457212_at | --- | 1.17 |  | 1419358_at | Sorcs2 | -1.08 |
| 1416786_at | Acvr1 | 1.14 |  | 1417323_at | Psrc1 | -1.06 |
| 1426812_a_at | Fam129b | 1.13 |  | 1430133_at | Tbc1d8b | -1.05 |
| 1428599_at | Kndc1 | 1.12 |  | 1436600_at | Tox3 | -1.04 |
| 1451474_a_at | Parp8 | 1.11 |  | 1421340_at | Map3k5 | -1.04 |
| 1456292_a_at | Vim | 1.11 |  | 1436037_at | Itga4 | -1.04 |
| 1448392_at | Sparc | 1.11 |  | 1423853_at | 6330527O06Rik | -1.04 |
| 1458126_at | D030041H20Rik | 1.10 |  | 1438483_at | Nos1 | -1.03 |
| 1460101_at | --- | 1.10 |  | 1423400_at | Kl | -1.03 |
| 1452244_at | 6330406I15Rik | 1.10 |  | 1457275_at | Synm | -1.03 |
| 1425963_at | Cabp7 | 1.10 |  | 1429905_at | Lhx9 | -1.02 |
| 1455346_at | Masp1 | 1.10 |  | 1445539_at | Pde7b | -1.02 |
| 1429906_at | A930035E12Rik | 1.09 |  | 1429022_at | Adcyap1r1 | -1.02 |
| 1459144_at | --- | 1.08 |  | 1429355_at | Tekt5 | -1.02 |
| 1455847_at | Pak7 | 1.08 |  | 1419874_x_at | Zbtb16 | -1.02 |
| 1438782_at | Cntn4 | 1.08 |  | 1435618_at | Pnma2 | -1.01 |
| 1416754_at | Prkar1b | 1.07 |  | 1449007_at | Btg3 /// Gm7334 | -1.01 |
| 1418402_at | Adam19 | 1.07 |  | 1436449_at | Pcdh11x | -1.01 |
| 1435367_at | Mapk4 | 1.06 |  | 1417680_at | Kcna5 | -1.00 |
| 1443115_at | --- | 1.06 |  | 1457183_at | Slc6a1 | -1.00 |
| 1460627_at | Thsd7b | 1.06 |  | 1416316_at | Slc27a2 | -0.98 |
| 1438193_at | Nrxn3 | 1.04 |  | 1429028_at | Dock11 | -0.98 |
| 1422818_at | Nedd9 | 1.04 |  | 1419230_at | Krt12 | -0.98 |
| 1450047_at | Hs6st2 | 1.04 |  | 1440209_at | 1-Mar | -0.98 |
| 1457361_at | Zfp804a | 1.03 |  | 1417489_at | Npy2r | -0.97 |
| 1440901_at | Dgkb | 1.03 |  | 1437382_at | Acvr2a | -0.96 |
| 1428562_at | 2210403K04Rik | 1.03 |  | 1455325_at | Miat | -0.96 |
| 1440358_at | Arhgef15 | 1.03 |  | 1438558_x_at | Foxq1 | -0.96 |
| 1427580_a_at | Rian | 1.02 |  | 1446282_at | LOC626082 | -0.96 |
| 1439019_at | Fras1 | 1.02 |  | 1455784_at | Sec1 | -0.95 |
| 1453008_at | Trnp1 | 1.02 |  | 1431905_s_at | 4933427G17Rik | -0.95 |
| 1443558_s_at | Nt5dc3 | 1.01 |  | 1422540_at | Fbln1 | -0.94 |
| 1439311_at | B830012L14Rik | 1.01 |  | 1416111_at | Cd83 | -0.94 |
| 1416155_at | Hmgb3 | 1.00 |  | 1448026_at | Chd7 | -0.93 |
| 1457551_at | --- | 1.00 |  | 1436657_at | Gm11744 | -0.93 |
| 1452590_a_at | Gm10393 /// Plac9 | 1.00 |  | 1460300_a_at | Ltk | -0.93 |
| 1437311_at | Snhg11 | 0.99 |  | 1458113_at | 9530019H20Rik | -0.92 |
| 1448260_at | Uchl1 | 0.99 |  | 1448327_at | Actn2 | -0.91 |
| 1449979_a_at | Spock3 | 0.98 |  | 1434049_at | Entpd3 | -0.91 |
| 1416811_s_at | Ctla2a /// Ctla2b | 0.97 |  | 1433893_s_at | Spag5 | -0.91 |
| 1451680_at | Srxn1 | 0.96 |  | 1439015_at | Gfra1 | -0.90 |
| 1418248_at | Gla | 0.96 |  | 1428301_at | ENSMUSG00000068790 /// Gm10128 /// Gm10406 /// Gm2897 /// Gm3373 /// Gm3558 /// Gm3696 | -0.90 |
| 1424542_at | S100a4 | 0.96 |  | 1418478_at | Lmo1 | -0.90 |
| 1433446_at | Hmgcs1 | 0.96 |  | 1422510_at | Ctdspl | -0.90 |
| 1457096_at | 6430520M22Rik /// Bhlhe41 | 0.95 |  | 1421137_a_at | Pkib | -0.89 |
| 1422520_at | Nefm | 0.95 |  | 1430253_at | 2900006B11Rik | -0.88 |
| 1455468_at | Dpy19l3 | 0.95 |  | 1420925_at | Tub | -0.88 |
| 1443302_at | 6720403M19Rik | 0.95 |  | 1417777_at | Ptgr1 | -0.87 |
| 1423754_at | Ifitm3 | 0.94 |  | 1452604_at | Stard13 | -0.87 |
| 1442001_at | Prkab2 | 0.94 |  | 1436585_at | BB182297 | -0.87 |
| 1458524_at | Fndc3a | 0.94 |  | 1456202_at | Elfn2 | -0.86 |
| 1421446_at | Prkcc | 0.94 |  | 1419420_at | St6galnac5 | -0.86 |
| 1415993_at | Sqle | 0.94 |  | 1415698_at | Golm1 | -0.86 |
| 1443006_at | --- | 0.93 |  | 1422930_at | Icam4 | -0.86 |
| 1429650_at | Stk40 | 0.93 |  | 1440557_at | Ipw | -0.86 |
| 1446316_at | Lpin2 | 0.93 |  | 1436493_at | Ctxn2 | -0.85 |
| 1454015_a_at | Cdh13 | 0.92 |  | 1440973_at | LOC552874 /// St6galnac5 | -0.85 |
| 1435880_at | Ankrd50 | 0.92 |  | 1451547_at | Iyd | -0.85 |
| 1426397_at | Tgfbr2 | 0.92 |  | 1436919_at | Trp53i11 | -0.84 |
| 1426818_at | Arrdc4 | 0.92 |  | 1456974_at | Onecut1 | -0.84 |
| 1435772_at | Kif21b | 0.91 |  | 1419070_at | Cys1 | -0.83 |
| 1434891_at | Ptgfrn | 0.91 |  | 1427509_at | Baiap3 | -0.83 |
| 1455642_a_at | Tspan17 | 0.91 |  | 1437360_at | Pcdh19 | -0.83 |
| 1425542_a_at | Ppp2r5c | 0.90 |  | 1456421_at | 4930578M01Rik | -0.83 |
| 1417893_at | Sfxn3 | 0.90 |  | 1453231_at | Cdkl1 | -0.83 |
| 1424694_at | 2010011I20Rik | 0.89 |  | 1429071_at | Me3 | -0.82 |
| 1438824_at | Slc20a1 | 0.89 |  | 1437876_at | Il20rb | -0.82 |
| 1458194_at | --- | 0.89 |  | 1452731_x_at | ENSMUSG00000068790 /// Gm10128 /// Gm2897 /// Gm3002 /// Gm3373 /// Gm3558 /// Gm3696 /// Gm8348 | -0.82 |
| 1446150_at | --- | 0.88 |  | 1448134_at | X99384 | -0.82 |
| 1432750_at | Zfp711 | 0.88 |  | 1441548_at | Frmd4b | -0.81 |
| 1417312_at | Dkk3 | 0.88 |  | 1437292_at | Clvs2 | -0.81 |
| 1451917_a_at | Dclk1 | 0.87 |  | 1429166_s_at | Clmn | -0.81 |
| 1460510_a_at | Coq10b | 0.87 |  | 1430096_at | 2900017F05Rik | -0.80 |
| 1451969_s_at | Parp3 | 0.87 |  | 1438672_at | Parvb | -0.80 |
| 1417051_at | Pcdh8 | 0.87 |  | 1427060_at | Mapk3 | -0.80 |
| 1416762_at | S100a10 | 0.87 |  | 1425396_a_at | Lck | -0.80 |
| 1417389_at | Gpc1 | 0.86 |  | 1439026_at | Trpm3 | -0.80 |
| 1422040_at | Sema7a | 0.86 |  | 1420342_at | Gdap10 | -0.79 |
| 1437681_at | Grik4 | 0.86 |  | 1425051_at | Isoc1 | -0.79 |
| 1435928_at | 6430548M08Rik | 0.86 |  | 1429254_at | Aqp11 | -0.79 |
| 1460440_at | Lphn3 | 0.86 |  | 1439189_at | Fnip2 | -0.79 |
| 1416430_at | Cat | 0.86 |  | 1446643_at | 5330409N07Rik | -0.79 |
| 1425382_a_at | Aqp4 | 0.85 |  | 1429758_at | 1700017B05Rik | -0.78 |
| 1422444_at | Itga6 | 0.84 |  | 1420911_a_at | Mfge8 | -0.78 |
| 1418492_at | Grem2 | 0.84 |  | 1435494_s_at | Dsp | -0.77 |
| 1424902_at | Plxdc1 | 0.84 |  | 1425615_a_at | Pck2 | -0.77 |
| 1439887_at | Rnf152 | 0.84 |  | 1416321_s_at | Prelp | -0.77 |
| 1456046_at | Cd93 | 0.84 |  | 1422747_at | Chek2 | -0.77 |
| 1417928_at | Pdlim4 | 0.84 |  | 1455410_at | Faim2 | -0.77 |
| 1459723_at | Zdhhc22 | 0.83 |  | 1447903_x_at | Ap1s2 | -0.77 |
| 1429240_at | Stard4 | 0.82 |  | 1438531_at | A730054J21Rik | -0.77 |
| 1420924_at | Timp2 | 0.81 |  | 1423878_at | Gypc | -0.76 |
| 1418329_at | Pgpep1 | 0.81 |  | 1434190_at | Sms | -0.76 |
| 1454745_at | Arhgap29 | 0.81 |  | 1453113_at | Wdsub1 | -0.76 |
| 1450683_at | Tagln3 | 0.81 |  | 1416251_at | Mcm6 | -0.76 |
| 1440128_s_at | Sez6l | 0.80 |  | 1455345_at | Phf15 | -0.76 |
| 1434069_at | Prex1 | 0.80 |  | 1418726_a_at | Tnnt2 | -0.76 |
| 1433741_at | Cd38 | 0.80 |  | 1422728_at | Inha | -0.76 |
| 1448503_at | Mcl1 | 0.80 |  | 1434754_at | Rap1gap2 | -0.75 |
| 1449111_a_at | Grb2 | 0.80 |  | 1423250_a_at | Tgfb2 | -0.75 |
| 1440831_at | Bach1 | 0.80 |  | 1426965_at | Rap2a | -0.75 |
| 1417435_at | Large | 0.80 |  | 1455754_at | Lmo3 | -0.75 |
| 1420981_a_at | Lmo4 | 0.79 |  | 1453250_at | Vwa3b | -0.75 |
| 1424229_at | Dyrk3 | 0.79 |  | 1435424_x_at | Shisa9 | -0.74 |
| 1441223_at | 4-Mar | 0.79 |  | 1418345_at | BC096441 /// Tnfsf13 | -0.74 |
| 1448613_at | Ecm1 | 0.79 |  | 1417937_at | Dact1 | -0.74 |
| 1430634_a_at | Pfkp | 0.79 |  | 1424882_a_at | Nt5dc2 | -0.74 |
| 1448130_at | Fdft1 | 0.79 |  | 1416511_a_at | Cdc42ep4 | -0.74 |
| 1426744_at | Srebf2 | 0.79 |  | 1426712_at | Slc6a15 | -0.74 |
| 1426617_a_at | Ttyh1 | 0.78 |  | 1442740_at | Prdm5 | -0.74 |
| 1457030_at | Mirg | 0.78 |  | 1428696_at | Rftn1 | -0.73 |
| 1449123_at | Itih3 | 0.78 |  | 1436395_at | Card6 | -0.73 |
| 1448471_a_at | Ctla2a | 0.78 |  | 1434838_at | Kcng2 | -0.73 |
| 1423584_at | Igfbp7 | 0.78 |  | 1438428_at | LOC100504883 | -0.72 |
| 1460206_at | Grasp | 0.78 |  | 1436624_at | Dnm3 | -0.72 |
| 1435551_at | Fhod3 | 0.78 |  | 1448647_at | Man2a1 | -0.72 |
| 1418892_at | Rhoj | 0.78 |  | 1439332_at | Ddit4l | -0.72 |
| 1448640_at | Slc14a1 | 0.77 |  | 1449411_at | Dscam | -0.72 |
| 1452646_at | Trp53inp2 | 0.77 |  | 1435438_at | Sox8 | -0.71 |
| 1435740_at | Gm10397 /// Zmiz1 | 0.77 |  | 1431109_at | Prr16 | -0.71 |
| 1442276_at | --- | 0.77 |  | 1442016_at | D630004N19Rik /// Mrpl48 | -0.71 |
| 1431094_at | 1110006E14Rik | 0.77 |  | 1439684_at | 4930570G19Rik | -0.71 |
| 1454551_at | 9530034D02Rik | 0.76 |  | 1419249_at | Cdk14 | -0.71 |
| 1455428_at | Fam53b | 0.76 |  | 1455618_x_at | Tspan33 | -0.71 |
| 1428765_at | Meg3 | 0.76 |  | 1448694_at | Jun | -0.71 |
| 1439174_at | --- | 0.76 |  | 1416368_at | Gsta4 | -0.71 |
| 1455946_x_at | Tmsb10 | 0.76 |  | 1438410_at | Prtg | -0.71 |
| 1455896_a_at | Kcnk1 | 0.76 |  | 1427338_at | Crocc | -0.71 |
| 1423693_at | Cela1 | 0.76 |  | 1456392_at | Negr1 | -0.71 |
| 1434564_at | E2f3 | 0.75 |  | 1460220_a_at | Csf1 | -0.70 |
| 1423078_a_at | Sc4mol | 0.75 |  | 1421508_at | Odz1 | -0.70 |
| 1460351_at | S100a11 | 0.74 |  | 1451321_a_at | Rbm43 | -0.70 |
| 1424529_s_at | Cgref1 | 0.74 |  | 1434539_at | Lrrn3 | -0.70 |
| 1448754_at | Rbp1 | 0.74 |  | 1456885_at | 8-Sep | -0.70 |
| 1451527_at | Pcolce2 | 0.74 |  | 1449491_at | Card10 | -0.70 |
| 1424796_at | 1700054N08Rik | 0.74 |  | 1417038_at | 9-Sep | -0.69 |
| 1418049_at | Ltbp3 | 0.74 |  | 1427427_at | Ryr3 | -0.69 |
| 1450646_at | Cyp51 | 0.74 |  | 1426934_at | Nhsl1 | -0.69 |
| 1455166_at | Arl5b | 0.74 |  | 1428372_at | St5 | -0.69 |
| 1439265_at | --- | 0.73 |  | 1417810_a_at | Kcnb1 /// Pacsin2 | -0.68 |
| 1450839_at | D0H4S114 | 0.73 |  | 1416997_a_at | Hap1 | -0.68 |
| 1440570_at | --- | 0.73 |  | 1450779_at | Fabp7 | -0.68 |
| 1423425_at | Plbd2 | 0.73 |  | 1454752_at | Rbm24 | -0.68 |
| 1452445_at | Slc41a2 | 0.73 |  | 1439773_at | Ly6e | -0.68 |
| 1452214_at | Skil | 0.73 |  | 1427086_at | Slit3 | -0.67 |
| 1430368_s_at | 1700019D03Rik | 0.72 |  | 1418461_at | Sh3d19 | -0.67 |
| 1434411_at | Col12a1 | 0.72 |  | 1426362_at | Tmem144 | -0.67 |
| 1458653_at | --- | 0.72 |  | 1448950_at | Il1r1 | -0.67 |
| 1419821_s_at | Idh1 | 0.71 |  | 1455401_at | Camkk2 | -0.67 |
| 1436614_at | --- | 0.71 |  | 1443847_x_at | Aff2 | -0.67 |
| 1421233_at | Pknox1 | 0.71 |  | 1421594_a_at | Sytl2 | -0.67 |
| 1449581_at | Emid1 | 0.71 |  | 1434141_at | Gucy1a3 | -0.67 |
| 1439986_at | Dgki | 0.71 |  | 1418183_a_at | Cyth1 | -0.66 |
| 1455216_at | Paqr6 | 0.71 |  | 1436277_at | Rnf207 | -0.66 |
| 1452490_a_at | Ap2a2 | 0.70 |  | 1427790_at | Adam1a | -0.66 |
| 1430135_at | Dnase2a | 0.70 |  | 1435959_at | Arhgap15 | -0.66 |
| 1417380_at | Iqgap1 | 0.70 |  | 1428332_at | Pik3ip1 | -0.66 |
| 1423816_at | Cxx1a /// Cxx1b | 0.69 |  | 1418285_at | Efnb1 | -0.66 |
| 1436584_at | Spry2 | 0.69 |  | 1455819_at | Rod1 | -0.66 |
| 1452066_a_at | Ndfip2 | 0.69 |  | 1453424_at | Fyco1 | -0.66 |
| 1419573_a_at | Lgals1 | 0.69 |  | 1427392_at | Dscaml1 | -0.66 |
| 1455140_at | Pitpnm3 | 0.69 |  | 1454979_at | Diap1 | -0.66 |
| 1416403_at | Abcb10 | 0.69 |  | 1434895_s_at | Ppp1r13b | -0.65 |
| 1450708_at | Scg2 | 0.69 |  | 1417505_s_at | Gm13305 /// Gm2002 /// Il11ra1 /// Il11ra2 | -0.65 |
| 1441554_at | Chsy3 | 0.68 |  | 1449237_at | Aloxe3 | -0.65 |
| 1435123_at | Efr3b | 0.68 |  | 1429718_at | Slitrk5 | -0.65 |
| 1441642_at | --- | 0.68 |  | 1439479_at | Lct | -0.64 |
| 1451201_s_at | Rnh1 | 0.68 |  | 1417304_at | Chrd | -0.64 |
| 1458811_at | 9430047L24Rik | 0.67 |  | 1436865_at | Slc26a11 | -0.64 |
| 1435518_at | Rap1b | 0.67 |  | 1443282_at | Prpf38a | -0.64 |
| 1451537_at | Chi3l1 | 0.67 |  | 1431569_a_at | Lypd1 | -0.64 |
| 1424581_at | Stac2 | 0.67 |  | 1430633_s_at | C430045I18Rik | -0.64 |
| 1436141_at | Cabp7 /// LOC100503092 | 0.66 |  | 1453059_at | 2310046A06Rik | -0.64 |
| 1447757_x_at | Inpp5f | 0.66 |  | 1455085_at | 1700086L19Rik | -0.64 |
| 1448551_a_at | Trim2 | 0.66 |  | 1427371_at | Abca8a | -0.64 |
| 1441172_at | --- | 0.66 |  | 1439707_at | C230034O21Rik | -0.64 |
| 1456816_at | --- | 0.66 |  | 1420477_at | Nap1l1 | -0.64 |
| 1440891_at | Gria4 | 0.66 |  | 1422048_at | Trpc5 | -0.64 |
| 1456339_at | 2810410D24Rik | 0.66 |  | 1457155_at | Aldh2 | -0.64 |
| 1450269_a_at | Pfkl | 0.66 |  | 1429270_a_at | Syce2 | -0.64 |
| 1436196_at | C030046G05 | 0.66 |  | 1433909_at | Syt17 | -0.64 |
| 1418498_at | Fgf13 | 0.65 |  | 1448745_s_at | Lor | -0.64 |
| 1427077_a_at | Ap2b1 | 0.65 |  | 1425212_a_at | Tnfrsf19 | -0.64 |
| 1443612_at | Ano3 | 0.65 |  | 1433907_at | Pknox2 | -0.63 |
| 1426534_a_at | Arfgap3 | 0.65 |  | 1426915_at | Dapk1 | -0.63 |
| 1416560_at | Slc13a3 | 0.65 |  | 1429089_s_at | 2900026A02Rik | -0.63 |
| 1421821_at | Ldlr | 0.65 |  | 1417772_at | Grhpr | -0.63 |
| 1426329_s_at | Baalc | 0.65 |  | 1452110_at | Mtrr | -0.63 |
| 1428643_at | Mgat5 | 0.65 |  | 1449423_at | Mast1 | -0.63 |
| 1435327_at | Lpgat1 | 0.64 |  | 1447896_s_at | Snhg8 | -0.63 |
| 1443365_at | Htr4 | 0.64 |  | 1438007_at | Fam19a2 | -0.63 |
| 1449056_at | E330009J07Rik | 0.64 |  | 1460192_at | Osbpl1a | -0.62 |
| 1421845_at | Golph3 | 0.64 |  | 1419032_at | 2610018G03Rik | -0.62 |
| 1456570_at | Epb4.1l4b | 0.63 |  | 1426743_at | Appl2 | -0.62 |
| 1437787_at | Lrrtm2 | 0.63 |  | 1440181_at | Gm1568 | -0.62 |
| 1449037_at | Crem | 0.63 |  | 1439806_at | Pogk | -0.62 |
| 1444482_at | A130078K24Rik | 0.63 |  | 1450253_a_at | Map3k4 | -0.62 |
| 1420816_at | Ywhag | 0.63 |  | 1450769_s_at | Stard5 | -0.62 |
| 1433939_at | Aff3 | 0.63 |  | 1429095_at | Cenpp | -0.62 |
| 1455192_at | Tmem198 | 0.63 |  | 1444107_at | C130039O16Rik | -0.62 |
| 1421654_a_at | Lmna | 0.62 |  | 1433945_at | Fam189a1 | -0.62 |
| 1445025_at | AU015536 | 0.62 |  | 1452332_at | Ccdc85a | -0.62 |
| 1416221_at | Fstl1 | 0.62 |  | 1423085_at | Efnb3 | -0.61 |
| 1439651_at | --- | 0.62 |  | 1429214_at | Adamtsl2 | -0.61 |
| 1421223_a_at | Anxa4 | 0.62 |  | 1452278_a_at | Hace1 | -0.61 |
| 1415823_at | Scd2 | 0.62 |  | 1433923_at | Krt77 | -0.61 |
| 1425131_at | Ptpn5 | 0.62 |  | 1434098_at | Glra2 | -0.61 |
| 1426510_at | Sccpdh | 0.62 |  | 1460003_at | AI956758 | -0.61 |
| 1426348_at | Col4a1 | 0.62 |  | 1424701_at | Pcdh20 | -0.61 |
| 1416382_at | Ctsc | 0.62 |  | 1454966_at | Itga8 | -0.60 |
| 1436917_s_at | Gpsm1 | 0.61 |  | 1452416_at | Il6ra | -0.60 |
| 1420514_at | Tmem47 | 0.61 |  | 1433795_at | Tgfbr3 | -0.60 |
| 1457163_at | D730035F11Rik | 0.61 |  | 1418445_at | Slc16a2 | -0.60 |
| 1448312_at | Pcsk2 | 0.61 |  | 1443187_at | Rspo3 | -0.60 |
| 1417179_at | Tspan5 | 0.61 |  | 1441931_x_at | Gss | -0.60 |
| 1430307_a_at | Me1 | 0.61 |  | 1436796_at | LOC100503380 /// Matr3 | -0.60 |
| 1449129_a_at | Kcnip3 | 0.61 |  | 1454821_at | B3gat1 | -0.60 |
| 1422637_at | Rassf5 | 0.61 |  | 1430068_at | C030011L09Rik | -0.59 |
| 1439397_at | Fmn1 | 0.60 |  | 1417101_at | Hspa2 | -0.59 |
| 1417033_at | Ube2g2 | 0.60 |  | 1449876_at | Prkg1 | -0.59 |
| 1418637_at | Etv3 | 0.60 |  | 1434655_at | Foxk1 | -0.59 |
| 1423526_at | Arid3b | 0.60 |  | 1452980_at | 2810468N07Rik | -0.59 |
| 1434199_at | Tmem151b | 0.60 |  | 1440343_at | Rps6ka5 | -0.59 |
| 1433325_at | 9330154F10Rik | 0.60 |  | 1449420_at | Pde1b | -0.59 |
| 1440698_at | --- | 0.60 |  | 1455040_s_at | Nhsl2 | -0.59 |
| 1448596_at | Slc6a8 | 0.60 |  | 1416623_at | Thbs3 | -0.59 |
| 1455016_at | Prpf38b | 0.60 |  | 1436998_at | Ankrd43 | -0.59 |
| 1426782_at | Gpr125 | 0.60 |  | 1418135_at | Aff1 | -0.59 |
| 1419819_s_at | Sec63 | 0.60 |  | 1429491_s_at | Rif1 | -0.59 |
| 1433605_at | Inpp5a | 0.60 |  | 1433885_at | Iqgap2 | -0.59 |
| 1426255_at | Nefl | 0.59 |  | 1452166_a_at | Krt10 | -0.59 |
| 1421017_at | Nrg3 | 0.59 |  | 1441963_at | Prosapip1 | -0.59 |
| 1427844_a_at | Cebpb | 0.59 |  | 1457558_at | A330050F15Rik | -0.58 |
| 1459497_at | --- | 0.59 |  | 1419113_at | Ap1g2 | -0.58 |
| 1453021_at | Stxbp5 | 0.59 |  | 1452696_a_at | 4933439C10Rik | -0.58 |
| 1423126_at | Atp1b3 | 0.59 |  | 1444232_at | LOC100503436 /// Prkg1 | -0.58 |
| 1425679_a_at | Mapk8ip1 | 0.59 |  | 1454822_x_at | Apcdd1 | -0.58 |
| 1457261_at | A930025H08Rik | 0.58 |  | 1437008_x_at | Tmem109 | -0.58 |
| 1448541_at | Klc1 | 0.58 |  | 1456487_at | Adcy1 | -0.58 |
| 1426670_at | Agrn | 0.58 |  | 1455161_at | AI504432 | -0.58 |
| 1416222_at | Nsdhl | 0.58 |  | 1434957_at | Cdon | -0.57 |
| 1441413_at | --- | 0.58 |  | 1420707_a_at | Traip | -0.57 |
| 1417128_at | Plekho1 | 0.58 |  | 1437265_at | 5330438D12Rik /// LOC100504423 | -0.57 |
| 1422612_at | Hk2 | 0.58 |  | 1437433_at | B3galt2 | -0.57 |
| 1436223_at | Itgb8 | 0.58 |  | 1429324_at | 1700012A16Rik | -0.56 |
| 1448957_at | Rbpj | 0.58 |  | 1455833_at | Afap1l2 | -0.56 |
| 1431645_a_at | Gdi2 | 0.58 |  | 1440350_at | Panx2 | -0.56 |
| 1437613_s_at | Ptpdc1 | 0.58 |  | 1451141_at | Mettl8 | -0.56 |
| 1448663_s_at | Mvd | 0.58 |  | 1456611_at | Fam13a | -0.56 |
| 1457682_at | Arhgap42 | 0.58 |  | 1460303_at | Nr3c1 | -0.56 |
| 1452656_at | Zdhhc2 | 0.57 |  | 1451534_at | Scgn | -0.56 |
| 1416642_a_at | Tpt1 | 0.57 |  | 1456662_at | AA386476 | -0.56 |
| 1449322_at | Gm13363 /// Ptp4a1 | 0.57 |  | 1433101_at | 9030419F21Rik | -0.55 |
| 1426719_at | Apbb2 | 0.57 |  | 1416498_at | Ppic | -0.55 |
| 1455025_at | Paqr9 | 0.57 |  | 1418369_at | Prim1 | -0.55 |
| 1436594_at | Zfp719 | 0.57 |  | 1428547_at | Nt5e | -0.55 |
| 1450744_at | Ell2 | 0.57 |  | 1441801_at | Kctd4 | -0.55 |
| 1435130_at | Fam18a | 0.56 |  | 1442659_at | Pcdh9 | -0.55 |
| 1454558_at | 5430416B10Rik | 0.56 |  | 1436059_at | Rfx1 | -0.55 |
| 1435016_at | Trak2 | 0.56 |  | 1453061_at | Elac1 | -0.55 |
| 1438450_at | Lin7a | 0.56 |  | 1416983_s_at | Foxo1 | -0.55 |
| 1423221_at | Tubb4 | 0.56 |  | 1426550_at | Sidt1 | -0.55 |
| 1426399_at | Vwa1 | 0.56 |  | 1448400_a_at | Smarcd2 | -0.55 |
| 1437147_at | Gabrg2 | 0.55 |  | 1425277_at | Slit1 | -0.55 |
| 1424847_at | Nefh | 0.55 |  | 1438033_at | Tef | -0.55 |
| 1425534_at | Stau2 | 0.55 |  | 1440534_at | 6330403A02Rik /// Gm10001 | -0.55 |
| 1421962_at | Dnajb5 | 0.55 |  | 1449921_s_at | Cpne6 | -0.55 |
| 1430726_at | Rassf8 | 0.55 |  | 1429658_a_at | Smc2 | -0.55 |
| 1421756_a_at | Gpr19 | 0.55 |  | 1455421_x_at | Fam131b | -0.55 |
| 1420833_at | Vamp2 | 0.55 |  | 1440215_at | Gm11818 | -0.54 |
| 1448671_at | Ube2e3 | 0.55 |  | 1417994_a_at | Accn1 | -0.54 |
| 1425301_at | Ncam2 | 0.54 |  | 1436582_at | Zdhhc15 | -0.54 |
| 1416825_at | Snta1 | 0.54 |  | 1422052_at | Cdh8 | -0.54 |
| 1418709_at | Cox7a1 | 0.54 |  | 1455110_at | Gabpb2 | -0.54 |
| 1418592_at | Dnaja4 | 0.54 |  | 1455074_at | Efcab1 | -0.54 |
| 1453063_at | Cltb | 0.54 |  | 1424037_at | Itpka | -0.54 |
| 1450007_at | 1500003O03Rik | 0.54 |  | 1449158_at | Kcnk2 | -0.54 |
| 1456411_at | Rccd1 | 0.54 |  | 1436464_at | LOC100504619 | -0.54 |
| 1434510_at | Papss2 | 0.54 |  | 1436275_at | Kcnip2 | -0.53 |
| 1420899_at | Rab18 | 0.53 |  | 1457759_at | A630081D01Rik | -0.53 |
| 1448707_at | Taf13 | 0.53 |  | 1455114_at | Ccno | -0.53 |
| 1417153_at | Nacc2 | 0.53 |  | 1455080_at | Ppp1r16b | -0.53 |
| 1418435_at | Mkrn1 | 0.53 |  | 1436011_at | Elmo2 | -0.53 |
| 1426108_s_at | Cacnb1 | 0.53 |  | 1446769_at | Ttc39c | -0.53 |
| 1437174_at | Tfdp2 | 0.53 |  | 1416148_at | Laptm4b | -0.53 |
| 1416013_at | Pld3 | 0.53 |  | 1449815_a_at | Ssbp2 | -0.53 |
| 1433864_at | Lrp12 | 0.53 |  | 1436085_at | Zbtb34 | -0.53 |
| 1420942_s_at | Rgs5 | 0.53 |  | 1436422_at | BC026590 | -0.52 |
| 1450099_a_at | Gba | 0.53 |  | 1439190_at | Fhad1 | -0.52 |
| 1420891_at | Wnt7b | 0.52 |  | 1436115_at | Gm266 | -0.52 |
| 1439266_a_at | Polr3k | 0.52 |  | 1456478_at | Pgm2l1 | -0.52 |
| 1448428_at | Nbl1 | 0.52 |  | 1421498_a_at | 2010204K13Rik | -0.52 |
| 1420977_at | Man1a2 | 0.52 |  | 1445550_at | Klhl32 | -0.52 |
| 1435653_at | Abhd2 | 0.52 |  | 1458915_at | C77949 | -0.52 |
| 1422959_s_at | Rnf114 | 0.52 |  | 1418894_s_at | Pbx2 | -0.52 |
| 1442347_at | Lrp8 | 0.52 |  | 1448678_at | Fam118a | -0.52 |
| 1435771_at | Plcb4 | 0.52 |  | 1436786_at | 1110069O07Rik | -0.51 |
| 1445297_at | Stxbp5l | 0.52 |  | 1456653_a_at | Mthfd1l | -0.51 |
| 1415989_at | Vcam1 | 0.52 |  | 1423146_at | Hes5 | -0.51 |
| 1418816_at | Chmp1b | 0.51 |  | 1458114_at | Samd12 | -0.51 |
| 1425567_a_at | Anxa5 | 0.51 |  | 1448181_at | Klf15 | -0.51 |
| 1426677_at | Flna | 0.51 |  | 1426412_at | Neurod1 | -0.51 |
| 1429060_at | Malat1 | 0.51 |  | 1434562_at | Mfap3 | -0.51 |
| 1421093_at | Slc7a10 | 0.51 |  | 1432745_at | 9430092D12Rik | -0.51 |
| 1419355_at | Klf7 | 0.51 |  | 1433756_at | S100pbp | -0.51 |
| 1435051_at | Wdr44 | 0.51 |  | 1455695_at | St8sia1 | -0.51 |
| 1446243_at | --- | 0.51 |  | 1435575_at | Kntc1 | -0.51 |
| 1421846_at | Wsb2 | 0.51 |  | 1458933_at | Slc22a15 | -0.51 |
| 1417428_at | Gng3 | 0.51 |  | 1457318_at | A330008L17Rik | -0.51 |
| 1435510_at | Ppm1h | 0.51 |  | 1430066_at | Fam184a | -0.51 |
| 1456733_x_at | Serpinh1 | 0.51 |  | 1456130_at | Thsd7a | -0.51 |
| 1419455_at | Il10rb | 0.50 |  | 1428146_s_at | Acaa2 | -0.51 |
| 1451457_at | Sc5d | 0.50 |  | 1420895_at | Tgfbr1 | -0.51 |
| 1422531_at | Syt5 | 0.50 |  | 1439331_at | 4932439E07Rik | -0.51 |
| 1448534_at | Sirpa | 0.50 |  | 1443904_at | Fads6 | -0.50 |
| 1425611_a_at | Cux1 | 0.50 |  | 1458176_at | Per3 | -0.50 |
| 1415828_a_at | Serp1 | 0.50 |  | 1436407_at | Fam123c | -0.50 |
| 1439292_at | --- | 0.50 |  | 1439281_at | Slc26a8 | -0.50 |
| 1451979_at | Kras | 0.50 |  | 1416711_at | Tbr1 | -0.50 |
| 1442068_at | LOC100504135 | 0.50 |  | 1436105_at | Ccdc74a | -0.50 |
| 1439573_at | Rtn4rl2 | 0.50 |  | 1418412_at | Tpd52l1 | -0.50 |
| 1438789_s_at | Dpysl3 | 0.50 |  | 1441302_at | LOC100502835 | -0.50 |
| 1454939_at | --- | 0.50 |  | 1449048_s_at | Rab4a | -0.50 |
| 1421786_at | Ppp3r1 | 0.50 |  | 1439496_at | Ston1 | -0.50 |
| 1427299_at | Rps6ka3 | 0.50 |  | 1456756_at | Zc3hav1l | -0.50 |
| 1422768_at | Syncrip | 0.50 |  | 1421836_at | Mtap7 | -0.49 |
| 1434882_at | Mtdh | 0.49 |  | 1423186_at | Tiam2 | -0.49 |
| 1417871_at | Hsd17b7 | 0.49 |  | 1448477_at | Chst12 | -0.49 |
| 1450147_at | Npcd /// Nptxr | 0.49 |  | 1449410_a_at | Gas5 | -0.49 |
| 1417412_at | F8a | 0.49 |  | 1416871_at | Adam8 | -0.49 |
| 1419601_at | Kcnj10 | 0.49 |  | 1416184_s_at | Hmga1 /// Hmga1-rs1 | -0.49 |
| 1419169_at | Mapk6 | 0.49 |  | 1451589_at | Gatsl2 | -0.49 |
| 1428652_at | 0610010F05Rik | 0.49 |  | 1454773_at | Rxra | -0.49 |
| 1434601_at | Amigo2 | 0.49 |  | 1435957_at | B830032F12 | -0.49 |
| 1447883_x_at | Map1lc3a | 0.49 |  | 1426729_at | Fam131a | -0.49 |
| 1418706_at | Slc38a3 | 0.49 |  | 1454857_at | Rnf122 | -0.49 |
| 1416653_at | Stxbp3a | 0.49 |  | 1435763_at | Tbc1d16 | -0.49 |
| 1434694_at | Lrrc8a | 0.49 |  | 1449799_s_at | Pkp2 | -0.49 |
| 1428082_at | Acsl5 | 0.49 |  | 1453670_at | Usp50 | -0.49 |
| 1423961_at | Wdr26 | 0.48 |  | 1426926_at | Plcg2 | -0.49 |
| 1449155_at | Polr3g | 0.48 |  | 1440990_at | Kif26b | -0.49 |
| 1449533_at | Tmem100 | 0.48 |  | 1422470_at | Bnip3 | -0.49 |
| 1432478_a_at | Rnf19b | 0.48 |  | 1447811_s_at | Amigo1 | -0.49 |
| 1430449_at | Kidins220 | 0.48 |  | 1456334_s_at | Zfp783 | -0.48 |
| 1420841_at | Ptprf | 0.48 |  | 1438979_s_at | 1700029I15Rik | -0.48 |
| 1437031_at | Acsl6 | 0.48 |  | 1418057_at | Tiam1 | -0.48 |
| 1426373_at | Ski | 0.48 |  | 1455584_at | Sdf4 | -0.48 |
| 1435542_s_at | Cttnbp2nl | 0.48 |  | 1429191_at | Dhx33 | -0.48 |
| 1428054_at | Slc8a2 | 0.48 |  | 1423802_at | Camkv | -0.48 |
| 1428354_at | Foxk2 | 0.48 |  | 1426584_a_at | Sord | -0.48 |
| 1418323_at | Fem1b | 0.48 |  | 1419488_at | Tnip2 | -0.48 |
| 1416700_at | Rnd3 | 0.48 |  | 1449510_at | Zfp467 | -0.48 |
| 1458230_at | --- | 0.48 |  | 1425006_a_at | Vrk1 | -0.48 |
| 1449590_a_at | Mras | 0.48 |  | 1435614_s_at | Rasgrf1 | -0.48 |
| 1454588_at | 9430006E15Rik | 0.47 |  | 1451499_at | Cadps2 | -0.48 |
| 1427061_at | Rbbp8 | 0.47 |  | 1434207_at | Rnf169 | -0.47 |
| 1417104_at | Emp3 | 0.47 |  | 1458183_at | 2810039B14Rik | -0.47 |
| 1415677_at | Dhrs1 | 0.47 |  | 1452840_at | 1500009L16Rik | -0.47 |
| 1438761_a_at | Odc1 | 0.47 |  | 1451268_at | Tram1l1 | -0.47 |
| 1420506_a_at | Stxbp1 | 0.47 |  | 1449279_at | Gpx2 | -0.47 |
| 1438780_at | Stx1b | 0.47 |  | 1426436_at | Tmem159 | -0.47 |
| 1416067_at | Ifrd1 | 0.47 |  | 1455460_at | Gm2612 | -0.47 |
| 1435646_at | Ikbkg | 0.47 |  | 1429955_at | 5031434O11Rik | -0.47 |
| 1427470_s_at | Napb | 0.47 |  | 1454685_at | Gpr146 | -0.47 |
| 1419024_at | Ptp4a1 | 0.47 |  | 1417701_at | Ppp1r14c | -0.47 |
| 1417018_at | Efemp2 | 0.47 |  | 1428052_a_at | Zmym1 | -0.47 |
| 1436205_at | Nfasc | 0.47 |  | 1419207_at | Zfp37 | -0.47 |
| 1428620_at | Ensa | 0.47 |  | 1442058_s_at | Psmc3ip | -0.47 |
| 1437347_at | Ednrb | 0.47 |  | 1418164_at | Stx2 | -0.46 |
| 1424954_a_at | Pip5k1c | 0.46 |  | 1447993_a_at | Snhg10 | -0.46 |
| 1456320_at | Fam126b | 0.46 |  | 1437298_at | Wipf2 | -0.46 |
| 1441481_at | Mfap3l | 0.46 |  | 1416948_at | Mrpl23 | -0.46 |
| 1416503_at | Lxn | 0.46 |  | 1457919_at | D030054H15Rik | -0.46 |
| 1422018_at | Hivep2 | 0.46 |  | 1417103_at | Ddt | -0.46 |
| 1426959_at | Bdh1 | 0.46 |  | 1435641_at | Mgat4a | -0.46 |
| 1437700_at | Schip1 | 0.46 |  | 1448299_at | Slc1a1 | -0.46 |
| 1456080_a_at | Serinc3 | 0.46 |  | 1430041_at | Snrnp35 | -0.46 |
| 1429384_at | Csnk1g3 | 0.46 |  | 1448246_at | Hdac1 | -0.46 |
| 1453744_a_at | Ankrd40 | 0.46 |  | 1450005_x_at | Dlk2 | -0.46 |
| 1453187_at | Ociad2 | 0.46 |  | 1452712_at | Hnrnpa3 | -0.46 |
| 1426754_x_at | Ckap4 | 0.46 |  | 1435627_x_at | Marcksl1 | -0.46 |
| 1451941_a_at | Fcgr2b | 0.46 |  | 1428434_at | Zcchc12 | -0.46 |
| 1422659_at | Camk2d | 0.46 |  | 1448125_at | Rit2 | -0.46 |
| 1418468_at | Anxa11 | 0.46 |  | 1453262_at | 2810032G03Rik | -0.46 |
| 1455425_at | Tet1 | 0.46 |  | 1436140_at | Fam116b | -0.46 |
| 1434902_at | Rnf157 | 0.46 |  | 1456606_a_at | Chst11 /// Phactr1 | -0.46 |
| 1422619_at | Ppap2a | 0.46 |  | 1434285_at | Frmd4a | -0.46 |
| 1415860_at | Kpna2 | 0.45 |  | 1449502_at | Dazl | -0.45 |
| 1415965_at | Scd1 | 0.45 |  | 1426893_at | Fam102a | -0.45 |
| 1439041_at | Slc39a10 | 0.45 |  | 1424544_at | Nrbp2 | -0.45 |
| 1449465_at | Reln | 0.45 |  | 1448919_at | Cd302 | -0.45 |
| 1452192_at | Naf1 | 0.45 |  | 1449270_at | Plxdc2 | -0.45 |
| 1436195_at | BC046404 | 0.45 |  | 1418317_at | Lhx2 | -0.45 |
| 1428578_s_at | Ppfia4 | 0.45 |  | 1417877_at | Eepd1 | -0.45 |
| 1441232_at | --- | 0.45 |  | 1441693_at | Adamts3 | -0.45 |
| 1436117_at | A830010M20Rik | 0.45 |  | 1438883_at | Fgf5 | -0.45 |
| 1429625_at | 2900054C01Rik | 0.45 |  | 1435260_at | Akt3 | -0.45 |
| 1428374_at | Glce | 0.45 |  | 1439633_at | Syt7 | -0.45 |
| 1447014_at | --- | 0.45 |  | 1457115_at | Gm10643 | -0.45 |
| 1460235_at | Scarb2 | 0.45 |  | 1439031_at | Jph4 | -0.45 |
| 1452227_at | Sel1l3 | 0.44 |  | 1421379_at | Zfp354b | -0.44 |
| 1416313_at | Mllt11 | 0.44 |  | 1435810_at | 5730455O13Rik | -0.44 |
| 1416289_at | Plod1 | 0.44 |  | 1454920_at | Uhrf2 | -0.44 |
| 1452204_at | Anks1 | 0.44 |  | 1455244_at | Daam1 | -0.44 |
| 1417818_at | Wwtr1 | 0.44 |  | 1455190_at | Gng7 | -0.44 |
| 1428623_at | Plxna1 | 0.44 |  | 1442830_at | Nusap1 | -0.44 |
| 1417192_at | Tomm70a | 0.44 |  | 1427261_at | Wwc1 | -0.44 |
| 1421491_a_at | Tmem49 | 0.44 |  | 1417903_at | Dfna5 | -0.44 |
| 1425076_at | Dnajc18 | 0.44 |  | 1446228_at | Ermp1 | -0.44 |
| 1453596_at | Id2 | 0.44 |  | 1456535_at | Vps13c | -0.44 |
| 1436124_at | Pcyt1b | 0.44 |  | 1436651_at | Prdm11 | -0.44 |
| 1437912_at | Iqsec3 | 0.44 |  | 1442525_at | Ccdc47 | -0.44 |
| 1419372_at | Gosr2 | 0.44 |  | 1437621_x_at | Phgdh | -0.44 |
| 1419499_at | Gpam | 0.44 |  | 1423563_at | Prrt1 | -0.44 |
| 1416748_a_at | Mre11a | 0.44 |  | 1439671_at | 4930466K18Rik | -0.44 |
| 1426053_a_at | Xpr1 | 0.43 |  | 1444082_at | A730017C20Rik | -0.44 |
| 1449348_at | Mpp6 | 0.43 |  | 1424253_at | Fam114a2 | -0.44 |
| 1429206_at | Rhobtb1 | 0.43 |  | 1435343_at | Dock10 | -0.44 |
| 1435598_at | LOC100503607 | 0.43 |  | 1454832_at | Phactr1 | -0.44 |
| 1438068_at | --- | 0.43 |  | 1418321_at | Dci | -0.43 |
| 1428637_at | Dyrk2 | 0.43 |  | 1418485_at | Slc4a3 | -0.43 |
| 1448269_a_at | Klhl13 | 0.43 |  | 1417820_at | Tor1b | -0.43 |
| 1425781_a_at | Plcb1 | 0.43 |  | 1443368_at | BB557941 | -0.43 |
| 1451679_at | 6530401D17Rik | 0.43 |  | 1454840_at | Mccc2 | -0.43 |
| 1421814_at | Msn | 0.43 |  | 1432944_at | 2900046L07Rik | -0.43 |
| 1421191_s_at | Gopc | 0.43 |  | 1449431_at | Trpc6 | -0.43 |
| 1451276_at | Uhrf1bp1l | 0.42 |  | 1441224_at | Drp2 | -0.43 |
| 1418749_at | Psd3 | 0.42 |  | 1457039_at | Cecr2 | -0.43 |
| 1449740_s_at | Dsg2 | 0.42 |  | 1455267_at | Esrrg | -0.43 |
| 1455139_at | Smarcal1 | 0.42 |  | 1453006_at | Fgfbp3 | -0.43 |
| 1422702_at | Azin1 | 0.42 |  | 1436845_at | Axin2 | -0.43 |
| 1427708_a_at | Nf2 | 0.42 |  | 1418400_at | Larp6 | -0.43 |
| 1435645_at | Mmd | 0.42 |  | 1460222_at | Sh3bp1 | -0.43 |
| 1434657_at | Gls | 0.42 |  | 1456901_at | Adamts20 | -0.43 |
| 1446029_at | --- | 0.42 |  | 1451336_at | Lgals4 | -0.43 |
| 1426648_at | Mapkapk2 | 0.42 |  | 1428964_at | Slc25a18 | -0.43 |
| 1423044_at | Prosc | 0.42 |  | 1454687_at | Lrfn5 | -0.43 |
| 1418825_at | Irgm1 | 0.41 |  | 1458913_at | 4831440E17Rik | -0.43 |
| 1426923_at | Agfg1 | 0.41 |  | 1456706_at | 4833441D16Rik | -0.43 |
| 1415987_at | Hdlbp | 0.41 |  | 1428638_at | Efhc2 | -0.43 |
| 1418296_at | Fxyd5 | 0.41 |  | 1422539_at | Extl2 | -0.43 |
| 1452290_at | Tmem106b | 0.41 |  | 1454858_x_at | Mettl7a1 | -0.43 |
| 1425481_at | Cnot6l | 0.41 |  | 1417972_s_at | Pop5 | -0.43 |
| 1446421_at | --- | 0.41 |  | 1436426_at | Cc2d2a | -0.42 |
| 1450214_at | Adora2b | 0.41 |  | 1435892_at | Asxl3 | -0.42 |
| 1443444_at | --- | 0.41 |  | 1437782_at | Cntnap2 | -0.42 |
| 1428986_at | Slc17a7 | 0.41 |  | 1456351_at | Brd8 | -0.42 |
| 1416303_at | Litaf | 0.41 |  | 1449027_at | Rhou | -0.42 |
| 1448992_at | Ina | 0.41 |  | 1447275_at | Bbs12 | -0.42 |
| 1420646_at | Nus1 | 0.41 |  | 1457133_at | Tcerg1l | -0.42 |
| 1429299_at | Ddah1 | 0.41 |  | 1452621_at | Pcbd2 | -0.42 |
| 1422764_at | Mapre1 | 0.40 |  | 1422580_at | Myl4 | -0.42 |
| 1416767_a_at | 1110003E01Rik | 0.40 |  | 1426422_at | Cc2d1a | -0.42 |
| 1427295_at | Slc38a10 | 0.40 |  | 1416795_at | Cryl1 | -0.42 |
| 1452981_at | Cntn1 | 0.40 |  | 1421918_at | Anp32a | -0.42 |
| 1417012_at | Sdc2 | 0.40 |  | 1455558_at | Plk1s1 | -0.42 |
| 1423642_at | Tubb2c | 0.40 |  | 1425264_s_at | Mbp | -0.42 |
| 1419655_at | Tle3 | 0.40 |  | 1435396_at | Stxbp6 | -0.42 |
| 1441895_x_at | --- | 0.40 |  | 1452832_s_at | Cds2 | -0.42 |
| 1451799_at | Ccdc25 | 0.40 |  | 1420996_at | Plxna3 | -0.42 |
| 1424981_at | Nln | 0.40 |  | 1452076_at | Spryd4 | -0.42 |
| 1426621_a_at | Ppp2r2b | 0.40 |  | 1435170_at | Tsr2 | -0.42 |
| 1434469_at | Otud4 | 0.40 |  | 1452973_at | Ppm1k | -0.41 |
| 1418972_at | Bcl10 | 0.40 |  | 1458985_at | Fry | -0.41 |
| 1425790_a_at | Grik2 | 0.40 |  | 1455397_at | Caskin1 | -0.41 |
| 1420868_s_at | Tmed2 | 0.40 |  | 1435609_at | Trp53bp1 | -0.41 |
| 1441691_at | --- | 0.40 |  | 1416760_at | Galntl1 | -0.41 |
| 1435419_at | Rgag4 | 0.40 |  | 1441127_at | Slitrk2 | -0.41 |
| 1431646_a_at | Stx6 | 0.39 |  | 1436596_at | H2afv | -0.41 |
| 1423557_at | Ifngr2 | 0.39 |  | 1417529_at | Rab33a | -0.41 |
| 1438707_at | Atp13a4 | 0.39 |  | 1439788_at | Ccdc111 | -0.41 |
| 1431802_a_at | Ept1 | 0.39 |  | 1451194_at | Aldob | -0.41 |
| 1433998_at | 4933427D14Rik | 0.39 |  | 1434261_at | Sipa1l2 | -0.41 |
| 1435820_x_at | Ddr1 | 0.39 |  | 1424379_at | Car11 | -0.41 |
| 1456422_at | Dennd5b | 0.39 |  | 1455095_at | Hist2h2be | -0.41 |
| 1427375_at | Rg9mtd2 | 0.39 |  | 1420913_at | Slco2a1 | -0.41 |
| 1448580_at | Glg1 | 0.39 |  | 1441022_at | Arih1 | -0.41 |
| 1416085_s_at | Zfand5 | 0.39 |  | 1421402_at | Mta3 | -0.41 |
| 1435472_at | Kremen1 | 0.39 |  | 1448700_at | G0s2 | -0.41 |
| 1441617_at | Med15 | 0.39 |  | 1424113_at | Lamb1 | -0.41 |
| 1420607_at | Rbm18 | 0.39 |  | 1434107_at | Spata2 | -0.41 |
| 1423493_a_at | Nfix | 0.39 |  | 1427974_s_at | Cacna1d | -0.41 |
| 1423824_at | Wls | 0.39 |  | 1424024_at | Mcfd2 | -0.41 |
| 1419243_at | Rab14 | 0.39 |  | 1428443_a_at | Rap1gap | -0.41 |
| 1430302_at | Cnrip1 | 0.39 |  | 1438915_at | 6720401G13Rik | -0.41 |
| 1423662_at | Atp6ap2 | 0.39 |  | 1438610_a_at | Cryz | -0.41 |
| 1445561_at | --- | 0.39 |  | 1427107_at | Slc16a11 | -0.41 |
| 1450414_at | Pdgfb | 0.39 |  | 1460738_at | Limd2 | -0.40 |
| 1434730_at | AI854517 | 0.39 |  | 1448498_at | Rps6ka4 | -0.40 |
| 1424093_x_at | Cd151 | 0.39 |  | 1455315_at | Trp53i13 | -0.40 |
| 1424538_at | Ubl4 | 0.38 |  | 1418158_at | Trp63 | -0.40 |
| 1448660_at | Arhgdig | 0.38 |  | 1426002_a_at | Cdc7 | -0.40 |
| 1433804_at | Jak1 | 0.38 |  | 1444046_at | Shisa7 | -0.40 |
| 1437197_at | Sorbs2 | 0.38 |  | 1434776_at | Sema5a | -0.40 |
| 1449145_a_at | Cav1 | 0.38 |  | 1416956_at | Kcnab2 | -0.40 |
| 1442309_at | --- | 0.38 |  | 1456069_at | Dtna | -0.40 |
| 1418181_at | Ptp4a3 | 0.38 |  | 1459415_at | Specc1 | -0.40 |
| 1433691_at | Ppp1r3c | 0.38 |  | 1449897_a_at | Mtcp1 | -0.40 |
| 1420548_a_at | 2310008H09Rik | 0.38 |  | 1424167_a_at | Pmm1 | -0.40 |
| 1421624_a_at | Enah | 0.38 |  | 1435062_at | Srrm4 | -0.40 |
| 1452170_at | Chpf2 | 0.38 |  | 1448245_at | Rpsa | -0.40 |
| 1459145_at | A930033H14Rik | 0.38 |  | 1431771_a_at | Irak1bp1 | -0.40 |
| 1449245_at | Grin2c | 0.38 |  | 1427496_at | Cep152 | -0.40 |
| 1452799_at | Fggy | 0.37 |  | 1451469_at | Cntln | -0.40 |
| 1452499_a_at | Kif2a | 0.37 |  | 1429189_at | Arsb | -0.40 |
| 1453257_at | Agpat5 | 0.37 |  | 1453847_at | A930037O16Rik | -0.40 |
| 1424482_at | Arhgef7 | 0.37 |  | 1451920_a_at | Rfc1 | -0.40 |
| 1426346_at | Prepl | 0.37 |  | 1450627_at | Ank | -0.40 |
| 1431170_at | Efna3 | 0.37 |  | 1456103_at | Pml | -0.40 |
| 1418058_at | Eltd1 | 0.37 |  | 1456950_at | Alms1 | -0.40 |
| 1420403_at | Atp2b2 | 0.37 |  | 1429264_at | C030044B11Rik | -0.40 |
| 1435902_at | Nudt18 | 0.37 |  | 1443163_at | Slc39a2 | -0.40 |
| 1434303_at | Raph1 | 0.37 |  | 1434592_at | Slc16a10 | -0.40 |
| 1423167_at | Mobkl3 | 0.37 |  | 1423104_at | Irs1 | -0.40 |
| 1433559_at | Slc45a4 | 0.37 |  | 1418789_at | Sntg2 | -0.39 |
| 1428544_at | 0610007L01Rik | 0.36 |  | 1435896_at | Sfxn2 | -0.39 |
| 1460393_a_at | Dusp7 | 0.36 |  | 1419260_a_at | Snrpb | -0.39 |
| 1450735_at | Pno1 | 0.36 |  | 1436935_x_at | Clns1a | -0.39 |
| 1454740_at | Mib1 | 0.36 |  | 1422925_s_at | Acot3 | -0.39 |
| 1448861_at | Traf5 | 0.36 |  | 1433811_at | Mllt6 | -0.39 |
| 1416491_at | Numbl | 0.36 |  | 1429052_at | Ptprd | -0.39 |
| 1449351_s_at | Pdgfc | 0.36 |  | 1428306_at | Ddit4 | -0.39 |
| 1417432_a_at | Gnb1 | 0.36 |  | 1425178_s_at | Shmt1 | -0.39 |
| 1417961_a_at | Trim30a | 0.36 |  | 1420704_at | Csf2ra /// LOC100045292 | -0.39 |
| 1430999_a_at | Scoc | 0.36 |  | 1456471_x_at | Gm6756 /// Phgdh | -0.39 |
| 1428416_at | Fam164a | 0.36 |  | 1455877_a_at | Nanos1 | -0.39 |
| 1453853_a_at | Arhgef12 | 0.36 |  | 1459911_at | Cdr2l | -0.39 |
| 1429589_at | Gad2 | 0.36 |  | 1434515_at | Ncoa1 | -0.39 |
| 1428799_at | Lca5 | 0.36 |  | 1457726_at | Rps15a | -0.39 |
| 1426375_s_at | Oxnad1 | 0.36 |  | 1418090_at | Plvap | -0.39 |
| 1451533_at | BC022687 | 0.36 |  | 1427544_a_at | Papola | -0.39 |
| 1435432_at | Agap1 | 0.36 |  | 1428466_at | Chd3 | -0.39 |
| 1415738_at | Txndc12 | 0.35 |  | 1415948_at | Creg1 | -0.39 |
| 1417847_at | Ulk2 | 0.35 |  | 1427012_at | Lancl1 | -0.39 |
| 1451008_at | St8sia3 | 0.35 |  | 1432713_at | 6430709C05Rik | -0.39 |
| 1443851_at | Ppp4r4 | 0.35 |  | 1440715_s_at | Cdkn2aipnl | -0.39 |
| 1419251_at | Eps15 | 0.35 |  | 1454735_at | Odf2 | -0.39 |
| 1431423_a_at | Med8 | 0.35 |  | 1455270_at | Adam11 | -0.39 |
| 1449256_a_at | Rab11a | 0.35 |  | 1417414_at | 3-Sep | -0.39 |
| 1452740_at | Myh10 | 0.35 |  | 1460363_at | Tnrc6c | -0.38 |
| 1460199_a_at | Pafah1b1 | 0.35 |  | 1436079_s_at | Vapb | -0.38 |
| 1457687_at | Bcl2 | 0.35 |  | 1435006_s_at | Abcb7 | -0.38 |
| 1426285_at | Lama2 | 0.35 |  | 1433832_at | Sun2 | -0.38 |
| 1416459_at | Arf2 | 0.35 |  | 1416687_at | Plod2 | -0.38 |
| 1434673_at | Gpr22 | 0.35 |  | 1455220_at | Frat2 | -0.38 |
| 1451218_at | Edem1 | 0.35 |  | 1416407_at | Pea15a | -0.38 |
| 1460617_s_at | Rab6b | 0.34 |  | 1426323_x_at | Siva1 | -0.38 |
| 1423936_at | Kctd5 | 0.34 |  | 1428886_at | Rbbp5 | -0.38 |
| 1424988_at | Mylip | 0.34 |  | 1438019_at | Ippk | -0.38 |
| 1431734_a_at | Dnajb4 | 0.34 |  | 1458305_at | Tmtc3 | -0.38 |
| 1416023_at | Fabp3 | 0.34 |  | 1444528_at | Zfp316 | -0.38 |
| 1437704_at | Mblac2 | 0.34 |  | 1453558_at | Efcab10 | -0.38 |
| 1449674_s_at | Pdcd6ip | 0.34 |  | 1435473_at | Gm347 | -0.38 |
| 1428268_at | Psd2 | 0.34 |  | 1449633_s_at | Nt5c3l | -0.38 |
| 1428129_at | Lman1 | 0.34 |  | 1427149_at | Plekha6 | -0.38 |
| 1448587_at | Tbc1d10a | 0.34 |  | 1455564_at | Bcr | -0.38 |
| 1430869_a_at | Habp4 | 0.34 |  | 1427275_at | Smc4 | -0.38 |
| 1432827_x_at | Ubc | 0.34 |  | 1436358_at | Atpaf1 | -0.38 |
| 1427114_at | Ttc19 | 0.34 |  | 1422614_s_at | Bloc1s1 /// Rdh5 | -0.38 |
| 1417763_at | Ssr1 | 0.34 |  | 1435045_s_at | Mapk8ip2 | -0.38 |
| 1425188_s_at | Sel1l | 0.34 |  | 1460247_a_at | Skp2 | -0.38 |
| 1416475_at | Ube2d2 | 0.34 |  | 1452915_at | Prkar2a | -0.38 |
| 1434381_at | Atmin | 0.34 |  | 1429595_at | 2700049A03Rik | -0.37 |
| 1428717_at | Scrn1 | 0.34 |  | 1428803_at | Acot6 | -0.37 |
| 1423283_at | Pitpna | 0.34 |  | 1460675_at | Igsf8 | -0.37 |
| 1436192_at | Arfgef2 | 0.34 |  | 1441880_x_at | Tmem149 | -0.37 |
| 1451422_at | Myo18a | 0.34 |  | 1457139_at | Auts2 | -0.37 |
| 1421729_a_at | Fert2 | 0.33 |  | 1435700_at | Tln2 | -0.37 |
| 1448200_at | Tcn2 | 0.33 |  | 1442116_at | Gpr176 | -0.37 |
| 1455480_s_at | Ube2d3 | 0.33 |  | 1448744_at | Galns | -0.37 |
| 1429634_at | Zfp580 | 0.33 |  | 1460561_x_at | Sepw1 | -0.37 |
| 1433974_at | Sephs1 | 0.33 |  | 1416842_at | Gstm5 | -0.37 |
| 1439614_at | --- | 0.33 |  | 1438665_at | Smpd3 | -0.37 |
| 1416480_a_at | Gm9790 /// Higd1a | 0.33 |  | 1424223_at | 1700020C11Rik | -0.37 |
| 1437763_at | Dcun1d3 | 0.33 |  | 1451040_at | Dtd1 | -0.37 |
| 1422751_at | Tle1 | 0.33 |  | 1435601_at | Phlpp2 | -0.37 |
| 1433509_s_at | Reep1 | 0.33 |  | 1435968_at | Ptprn2 | -0.37 |
| 1416119_at | Txn1 | 0.33 |  | 1460564_at | Zfp280b | -0.37 |
| 1417966_at | Mrpl39 | 0.33 |  | 1456336_at | Csrnp3 | -0.37 |
| 1451208_at | Etf1 | 0.33 |  | 1435554_at | Tmcc3 | -0.37 |
| 1447946_at | Adam23 | 0.33 |  | 1416816_at | Nek7 | -0.37 |
| 1452883_a_at | Ncrna00081 | 0.33 |  | 1420127_s_at | Ccpg1 /// Dyx1c1 | -0.37 |
| 1418299_at | Dpysl4 | 0.33 |  | 1429058_at | Tmem107 | -0.37 |
| 1448438_at | Derl2 | 0.33 |  | 1448494_at | Gas1 | -0.37 |
| 1438740_at | Nmt2 | 0.33 |  | 1428240_at | Nrxn1 | -0.37 |
| 1422593_at | Ap3s1 | 0.32 |  | 1419186_a_at | St8sia4 | -0.37 |
| 1460041_at | Flrt1 | 0.32 |  | 1460434_at | Fundc2 | -0.37 |
| 1448302_at | Kctd20 | 0.32 |  | 1417371_at | Peli1 | -0.37 |
| 1439018_at | Fhdc1 | 0.32 |  | 1416416_x_at | Gstm1 | -0.37 |
| 1424384_a_at | Znrf1 | 0.32 |  | 1434337_at | Gm9853 | -0.37 |
| 1448475_at | Olfml3 | 0.32 |  | 1417393_a_at | Fam132a | -0.36 |
| 1425929_a_at | Rnf14 | 0.32 |  | 1429792_at | Btbd19 | -0.36 |
| 1452779_at | Ube2ql1 | 0.32 |  | 1437071_at | Eif1ax | -0.36 |
| 1453915_a_at | Slc37a3 | 0.32 |  | 1428401_at | Zcchc3 | -0.36 |
| 1452338_s_at | Itsn1 | 0.32 |  | 1453078_at | 2610002M06Rik | -0.36 |
| 1443205_at | D5Buc30e | 0.32 |  | 1436244_a_at | Tle2 | -0.36 |
| 1422009_at | Atp1b2 | 0.32 |  | 1455070_at | Dcp2 | -0.36 |
| 1426736_at | Gspt1 | 0.32 |  | 1427082_at | Sdr42e1 | -0.36 |
| 1428572_at | Basp1 | 0.32 |  | 1434083_a_at | Elmod1 | -0.36 |
| 1451047_at | Itm2a | 0.32 |  | 1417190_at | Nampt | -0.36 |
| 1428948_at | Kcnma1 | 0.32 |  | 1435391_at | Rbm33 | -0.36 |
| 1415956_a_at | Cdk16 | 0.31 |  | 1441867_x_at | 4930534B04Rik | -0.36 |
| 1421349_x_at | Cend1 | 0.31 |  | 1435085_at | Crebl2 | -0.36 |
| 1455684_at | Map3k9 | 0.31 |  | 1454967_at | A930001N09Rik | -0.36 |
| 1427933_at | Vps33b | 0.31 |  | 1425668_a_at | St3gal4 | -0.36 |
| 1451308_at | Elovl4 | 0.31 |  | 1416122_at | Ccnd2 | -0.36 |
| 1423053_at | Arf4 | 0.31 |  | 1456087_at | Nfia | -0.36 |
| 1436306_at | Ppp6r1 | 0.31 |  | 1425968_s_at | Speg | -0.36 |
| 1452274_at | Zfand3 | 0.31 |  | 1430530_s_at | Nmral1 | -0.36 |
| 1453581_at | Cep170 | 0.31 |  | 1435795_at | Glb1 | -0.36 |
| 1428124_at | Gtf2e1 | 0.31 |  | 1455381_at | 4921513D23Rik | -0.36 |
| 1438157_s_at | Nfkbia | 0.30 |  | 1454879_s_at | 1700052N19Rik | -0.36 |
| 1425575_at | Epha3 | 0.30 |  | 1453141_at | 0610009L18Rik | -0.36 |
| 1453035_at | Lnp | 0.30 |  | 1425662_at | Cdadc1 | -0.36 |
| 1450729_at | Hs2st1 | 0.30 |  | 1430158_at | 3110021A11Rik | -0.36 |
| 1425822_a_at | Dtx1 | 0.30 |  | 1429108_at | Msl2 | -0.36 |
| 1427162_a_at | Elk4 | 0.30 |  | 1433180_at | Morn1 | -0.36 |
| 1417062_at | Armc10 | 0.30 |  | 1435666_at | Mast3 | -0.35 |
| 1433338_at | 6720460K10Rik | 0.30 |  | 1451676_at | Drap1 | -0.35 |
| 1418046_at | Nap1l2 | 0.30 |  | 1459840_s_at | Ccdc28b | -0.35 |
| 1428333_at | 2900062L11Rik | 0.30 |  | 1426760_at | Ipo8 | -0.35 |
| 1428413_at | Ccny | 0.30 |  | 1426983_at | Fnbp1 | -0.35 |
| 1448348_at | Caprin1 | 0.30 |  | 1417220_at | Fah | -0.35 |
| 1452057_at | Actr1b | 0.30 |  | 1440202_at | E430024I08Rik | -0.35 |
| 1450845_a_at | Bzw1 | 0.29 |  | 1416958_at | Nr1d2 | -0.35 |
| 1450870_at | Rala | 0.29 |  | 1419350_at | Hook2 | -0.35 |
| 1418953_at | Fbxo16 | 0.29 |  | 1428454_at | Bcas3 | -0.35 |
| 1416935_at | Trpv2 | 0.29 |  | 1448600_s_at | Vav3 | -0.35 |
| 1430519_a_at | Cnot7 | 0.29 |  | 1458208_s_at | Mccc1 | -0.35 |
| 1434398_at | Nkrf | 0.29 |  | 1428291_at | Exosc8 | -0.35 |
| 1428510_at | Lphn1 | 0.29 |  | 1452715_at | Haus5 | -0.35 |
| 1435136_at | Whsc1 | 0.29 |  | 1437872_at | Napepld | -0.35 |
| 1453163_at | Ppp1r12a | 0.29 |  | 1437159_at | Tmppe | -0.35 |
| 1433802_at | Tmem151a | 0.29 |  | 1448016_at | Sass6 | -0.35 |
| 1433703_s_at | Bahd1 | 0.29 |  | 1449316_at | Cyp4f15 | -0.35 |
| 1420500_at | Dnajc1 | 0.29 |  | 1435776_at | 2310009B15Rik | -0.35 |
| 1448603_at | Srpk2 | 0.28 |  | 1426630_at | Exosc2 | -0.35 |
| 1425384_a_at | Ube4a | 0.28 |  | 1456946_at | Sh3rf3 | -0.35 |
| 1454043_a_at | Kcnab1 | 0.28 |  | 1438198_at | Bri3bp | -0.35 |
| 1434312_at | Arf6 | 0.28 |  | 1435452_at | Tmem20 | -0.35 |
| 1437111_at | Zc3h12c | 0.28 |  | 1448493_at | Paip2 | -0.35 |
| 1448748_at | Plek | 0.28 |  | 1428552_at | 2610001J05Rik | -0.34 |
| 1433587_at | Rgmb | 0.28 |  | 1457585_at | C2cd2l | -0.34 |
| 1424346_at | Ppp6c | 0.28 |  | 1416052_at | Prps1 | -0.34 |
| 1448499_a_at | Ephx2 | 0.28 |  | 1453413_at | Gnas | -0.34 |
| 1450662_at | Tesk1 | 0.27 |  | 1442562_at | Zfp827 | -0.34 |
| 1439612_at | Cacna1b | 0.27 |  | 1449118_at | Dbt | -0.34 |
| 1453015_at | 4933407C03Rik | 0.27 |  | 1436215_at | Ipmk | -0.34 |
|  |  |  |  | 1435706_at | Btbd9 | -0.34 |
|  |  |  |  | 1454959_s_at | Gnai1 | -0.34 |
|  |  |  |  | 1452848_at | Tmem181a /// Tmem181b-ps | -0.34 |
|  |  |  |  | 1415932_x_at | Atp9a | -0.34 |
|  |  |  |  | 1416526_a_at | Park7 | -0.34 |
|  |  |  |  | 1419903_at | Dbndd2 | -0.34 |
|  |  |  |  | 1435900_at | Zbtb43 | -0.34 |
|  |  |  |  | 1449001_at | Ivd | -0.34 |
|  |  |  |  | 1430151_at | Nisch | -0.34 |
|  |  |  |  | 1429779_at | Eif2c4 | -0.34 |
|  |  |  |  | 1454837_at | Cln6 | -0.34 |
|  |  |  |  | 1452682_at | Fam122b | -0.33 |
|  |  |  |  | 1440851_at | Erlec1 | -0.33 |
|  |  |  |  | 1442024_at | Ppp1r3e | -0.33 |
|  |  |  |  | 1436884_x_at | Ewsr1 | -0.33 |
|  |  |  |  | 1456259_at | Ints10 | -0.33 |
|  |  |  |  | 1438051_at | Ttc14 | -0.33 |
|  |  |  |  | 1428731_at | Usp54 | -0.33 |
|  |  |  |  | 1460007_at | Thyn1 | -0.33 |
|  |  |  |  | 1435827_at | 4933404O12Rik | -0.33 |
|  |  |  |  | 1448972_at | Gria1 | -0.33 |
|  |  |  |  | 1423522_at | Npm3 /// Npm3-ps1 | -0.33 |
|  |  |  |  | 1452497_a_at | Nfatc3 | -0.33 |
|  |  |  |  | 1434326_x_at | Coro2b | -0.33 |
|  |  |  |  | 1446719_at | Atad2b | -0.33 |
|  |  |  |  | 1429612_at | Eml4 | -0.32 |
|  |  |  |  | 1416850_s_at | Cisd1 | -0.32 |
|  |  |  |  | 1455313_at | Ablim2 | -0.32 |
|  |  |  |  | 1458003_at | Zfp398 | -0.32 |
|  |  |  |  | 1428854_at | Tmed8 | -0.32 |
|  |  |  |  | 1424442_a_at | Pja2 | -0.32 |
|  |  |  |  | 1452659_at | Dek | -0.32 |
|  |  |  |  | 1437853_x_at | Ndn | -0.32 |
|  |  |  |  | 1441870_s_at | Pkd2 | -0.32 |
|  |  |  |  | 1436663_at | AI846148 | -0.32 |
|  |  |  |  | 1429557_at | Mcm8 | -0.32 |
|  |  |  |  | 1450835_a_at | Gfra4 | -0.32 |
|  |  |  |  | 1418546_a_at | Stambpl1 | -0.32 |
|  |  |  |  | 1424433_at | Msrb2 | -0.32 |
|  |  |  |  | 1449243_a_at | Rps19 | -0.32 |
|  |  |  |  | 1430258_at | 2810422J05Rik | -0.32 |
|  |  |  |  | 1434781_at | Dnajc16 | -0.32 |
|  |  |  |  | 1452833_at | Rapgef2 | -0.32 |
|  |  |  |  | 1457690_at | Kalrn | -0.32 |
|  |  |  |  | 1454987_a_at | H2-Ke6 | -0.32 |
|  |  |  |  | 1423371_at | Pole4 | -0.32 |
|  |  |  |  | 1428866_at | 2810037O22Rik | -0.32 |
|  |  |  |  | 1439934_at | Slc30a10 | -0.32 |
|  |  |  |  | 1439754_at | Sox12 | -0.32 |
|  |  |  |  | 1455064_at | Rab36 | -0.31 |
|  |  |  |  | 1420824_at | Sema4d | -0.31 |
|  |  |  |  | 1416453_x_at | Gm10063 /// Rps12 /// Rps12-ps2 | -0.31 |
|  |  |  |  | 1425311_at | 4930432F04Rik | -0.31 |
|  |  |  |  | 1454843_at | Prps2 | -0.31 |
|  |  |  |  | 1433882_at | Cnot10 | -0.31 |
|  |  |  |  | 1448743_at | Ssx2ip | -0.31 |
|  |  |  |  | 1427020_at | Scara3 | -0.31 |
|  |  |  |  | 1451234_at | Fam193b | -0.31 |
|  |  |  |  | 1451229_at | Hdac11 | -0.31 |
|  |  |  |  | 1455177_at | Ahi1 | -0.31 |
|  |  |  |  | 1436616_at | Inpp4a | -0.31 |
|  |  |  |  | 1460135_at | A930005H10Rik | -0.31 |
|  |  |  |  | 1449415_at | Chd1l | -0.31 |
|  |  |  |  | 1438835_a_at | Eftud2 | -0.31 |
|  |  |  |  | 1438233_at | Fam178a | -0.31 |
|  |  |  |  | 1434653_at | Ptk2b | -0.31 |
|  |  |  |  | 1427535_s_at | Obsl1 | -0.31 |
|  |  |  |  | 1434620_s_at | Fam13b | -0.31 |
|  |  |  |  | 1432686_at | 4833406M21Rik | -0.31 |
|  |  |  |  | 1416712_at | Pepd | -0.31 |
|  |  |  |  | 1436096_at | Mkln1 | -0.31 |
|  |  |  |  | 1429537_at | Sfrs18 | -0.31 |
|  |  |  |  | 1436134_at | Scn2b | -0.31 |
|  |  |  |  | 1452209_at | Pkp4 | -0.31 |
|  |  |  |  | 1427342_at | Fastkd1 | -0.31 |
|  |  |  |  | 1428763_at | Smug1 | -0.30 |
|  |  |  |  | 1452464_a_at | Metap1d | -0.30 |
|  |  |  |  | 1438626_x_at | Rpl14 | -0.30 |
|  |  |  |  | 1418128_at | Adcy6 | -0.30 |
|  |  |  |  | 1460406_at | Pls1 | -0.30 |
|  |  |  |  | 1456856_at | Ppfia2 | -0.30 |
|  |  |  |  | 1437350_at | Ovca2 | -0.30 |
|  |  |  |  | 1443640_at | Zfp882 | -0.30 |
|  |  |  |  | 1447521_x_at | Arhgap39 | -0.30 |
|  |  |  |  | 1419645_at | Cstf2 | -0.30 |
|  |  |  |  | 1455680_at | 9630025H16Rik /// Kpna4 | -0.30 |
|  |  |  |  | 1449236_at | Dll3 | -0.30 |
|  |  |  |  | 1451326_at | Abhd14b | -0.30 |
|  |  |  |  | 1428281_at | Trub1 | -0.30 |
|  |  |  |  | 1451592_at | Mypop | -0.29 |
|  |  |  |  | 1457669_x_at | Rfc2 | -0.29 |
|  |  |  |  | 1455703_at | Akt2 | -0.29 |
|  |  |  |  | 1434129_s_at | Lhfpl2 | -0.29 |
|  |  |  |  | 1434771_at | 0610011F06Rik | -0.29 |
|  |  |  |  | 1418100_at | A030009H04Rik | -0.29 |
|  |  |  |  | 1423888_at | Telo2 | -0.29 |
|  |  |  |  | 1435218_at | Rasgef1a | -0.29 |
|  |  |  |  | 1450699_at | Selenbp1 | -0.29 |
|  |  |  |  | 1435547_at | Mkl2 | -0.29 |
|  |  |  |  | 1437570_at | AI503301 | -0.29 |
|  |  |  |  | 1453342_at | Cdc40 | -0.28 |
|  |  |  |  | 1417854_at | Map2k5 | -0.28 |
|  |  |  |  | 1428213_at | Nsmce4a | -0.28 |
|  |  |  |  | 1424516_at | B230354K17Rik | -0.28 |
|  |  |  |  | 1452933_at | Ankrd39 | -0.28 |
|  |  |  |  | 1434859_at | Umps | -0.27 |
|  |  |  |  | 1416198_at | Th1l | -0.27 |
|  |  |  |  | 1438224_at | Zswim5 | -0.27 |
|  |  |  |  | 1417800_at | Parp2 | -0.27 |
|  |  |  |  | 1417582_s_at | Dhodh | -0.27 |
